# The mechanism of RNA capping by SARS-CoV-2

**DOI:** 10.21203/rs.3.rs-1336910/v1

**Published:** 2022-02-15

**Authors:** Gina J. Park, Adam Osinski, Genaro Hernandez, Jennifer L. Eitson, Abir Majumdar, Marco Tonelli, Katie Henzler-Wildman, Krzysztof Pawłowski, Zhe Chen, Yang Li, John W. Schoggins, Vincent S. Tagliabracci

**Affiliations:** 1 Department of Molecular Biology, University of Texas Southwestern Medical Center, Dallas, TX 75390, USA.; 2 Department of Microbiology, University of Texas Southwestern Medical Center, Dallas, TX 75390, USA.; 3 Department of Biochemistry, University of Wisconsin-Madison, Madison, WI 53706, USA.; 4 Department of Biochemistry and Microbiology, Institute of Biology, Warsaw University of Life Sciences, Warsaw 02-776, Poland.; 5 Department of Biophysics, University of Texas Southwestern Medical Center, Dallas, TX 75390, USA.; 6 Harold C. Simmons Comprehensive Cancer Center, University of Texas Southwestern Medical Center, Dallas, Texas 75390, USA; 7 Hamon Center for Regenerative Science and Medicine, University of Texas Southwestern Medical Center, Dallas, Texas 75390, USA; 8 Howard Hughes Medical Institute, University of Texas Southwestern Medical Center, Dallas, Texas 75390, USA

## Abstract

The SARS-CoV-2 RNA genome contains a 5′-cap that facilitates translation of viral proteins, protection from exonucleases and evasion of the host immune response^1-4^. How this cap is made is not completely understood. Here, we reconstitute the SARS-CoV-2 ^7Me^GpppA_2′-O-Me_-RNA cap using virally encoded non-structural proteins (nsps). We show that the kinase-like NiRAN domain^5^ of nsp12 transfers RNA to the amino terminus of nsp9, forming a covalent RNA-protein intermediate (a process termed RNAylation). Subsequently, the NiRAN domain transfers RNA to GDP, forming the cap core structure GpppA-RNA. The nsp14^6^ and nsp16^7^ methyltransferases then add methyl groups to form functional cap structures. Structural analyses of the replication-transcription complex bound to nsp9 identified key interactions that mediate the capping reaction. Furthermore, we demonstrate in a reverse genetics system^8^ that the N-terminus of nsp9 and the kinase-like active site residues in the NiRAN domain are required for successful SARS-CoV-2 replication. Collectively, our results reveal an unconventional mechanism by which SARS-CoV-2 caps its RNA genome, thus exposing a new target in the development of antivirals to treat COVID-19.

Coronaviruses (CoVs) are a family of positive-sense, single-stranded RNA viruses that cause disease in humans, ranging from mild common colds to more severe respiratory infections^9^. The most topical of these is severe acute respiratory syndrome coronavirus 2 (SARS-CoV-2), the etiological agent of the ongoing COVID-19 pandemic, which to date has resulted in over 5.7-million deaths and almost 400-million cases globally^10^.

The SARS-CoV-2 RNA genome contains two open reading frames (ORF1a and ORF1ab), which are translated by host ribosomes to form two large polyproteins^2^. These polyproteins are subsequently cleaved by viral proteases to form 16 non-structural proteins (nsp1-16), some of which make up the Replication-Transcription Complex (RTC)^2^. At the core of the RTC is the nsp12 RNA-dependent RNA polymerase (RdRp), which is the target of several promising antivirals used to treat COVID-19 including remdesivir^11^ and molnupiravir^12^. In addition to the RdRp domain, nsp12 contains an N-terminal Nidovirus RdRp-Associated Nucleotidyltransferase (NiRAN) domain (Fig. 1a)^5^. The NiRAN domain shares sequence and structural similarity with the pseudokinase selenoprotein-O (SelO), which transfers AMP from ATP to protein substrates (a process termed AMPylation)^13-15^. Notably, the active site kinase-like residues of the NiRAN domain are highly conserved in *Nidovirales* (Extended Data Fig. 1) and are required for equine arteritis virus (EAV) and SARS-CoV-1 replication in cell culture^5^. Several hypotheses for the function of the NiRAN domain have been proposed, including roles in protein-primed RNA synthesis, RNA ligation, and mRNA capping^5,16^.

The CoV RNA genome, like eukaryotic mRNAs, contains a methylated guanosine linked to the first nucleotide of the RNA via a reverse 5′ to 5′ triphosphate linkage (Extended Data Fig. 2)^1,4^. This 5′ cap is important for RNA stability, initiation of mRNA translation, and protection from exonucleases^17^. Methylation of the ribose 2′-OH position of the first nucleotide completes the cap and protects the RNA from the host immune system^18,19^. Thus, formation of the RNA cap is crucial for successful replication and transcription of the viral genome.

All eukaryotes share a conserved co-transcriptional capping mechanism (Extended Data Fig. 2) involving: **1)** an RNA triphosphatase (RTPase), which removes the γ-phosphate from the nascent 5′-triphosphorylated RNA (5′-pppRNA) to yield a 5′-diphosphorylated RNA (5′-ppRNA); **2)** a guanylyltransferase (GTase), which transfers GMP from GTP to 5′-ppRNA to form the cap core structure GpppN-RNA; **3)** a (guanine-N7)-methyltransferase (N7-MTase), which methylates the cap guanine at the N7 position; and **4)** a (nucleoside-2′-*O*)-methyltransferase (2′-O-MTase), which methylates the ribose-2′-OH position on the first nucleotide of the RNA. In CoVs, the nsp13, nsp14, and nsp16 proteins have RTPase^20^, N7-MTase^6^, and 2′-O-MTase^7^ activities, respectively. Thus, it was presupposed that the CoV capping mechanism occurs in a similar fashion to the eukaryotic capping pathway, with the NiRAN domain functioning as the GTase^3,5,21^. However, evidence to support this claim has been lacking.

In this study, we discover that the NiRAN domain transfers monophosphorylated RNA (5′-pRNA) from 5′-pppRNA to the N-terminus of nsp9 as an intermediate step in cap synthesis. The NiRAN domain then transfers 5′-pRNA from RNAylated nsp9 to GDP to form the cap core structure GpppA-RNA. We then reconstitute cap-0 and cap-1 structures using the nsp14 and nsp16 methyltransferases. Furthermore, we present a cryo-EM structure of the SARS-CoV-2 RTC with the native N-terminus of nsp9 bound in the NiRAN active site. Finally, we demonstrate in a reverse genetics system that the N-terminus of nsp9 and the kinase-like active site residues in the NiRAN domain are required for SARS-CoV-2 replication.

## The NiRAN domain NMPylates the N-terminus of nsp9

**Figure 1.**
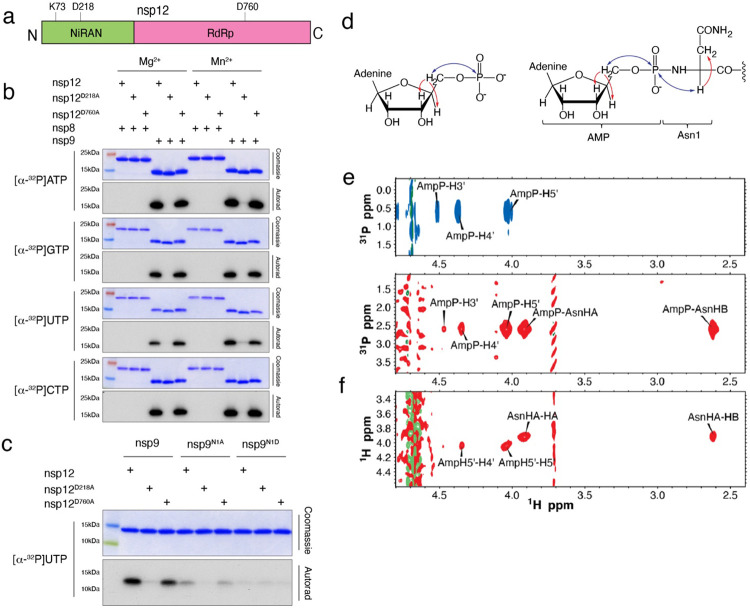
The NiRAN domain NMPylates the N-terminus of nsp9. **a.** Domain architecture of nsp12 depicting the SelO -like NiRAN domain (*green)* and the RNA-dependent RNA polymerase domain (RdRp; *magenta*), annotated with the predicted catalytic residues. **b.** Incorporation of α-^32^P from [α-^32^P]ATP, GTP, UTP, or CTP into nsp8 or nsp9 by WT nsp12, the NiRAN mutant (D218A), or the polymerase mutant (D760A). Reactions were performed in the presence of Mg^2+^ or Mn^2+^ and the products were resolved by SDS-PAGE and visualized by Coomassie staining (*top*) and autoradiography (*bottom*). **c.** Incorporation of α-^32^P from [α-^32^P]UTP into nsp9 or the indicated mutants by the NiRAN domain. Reaction products were analysed as in **b**. **d.** Structure of AMP (*left)* and AMP-nsp9 (*right*) with arrows to indicate the magnetization transfer steps that result in the peaks observed in the 2D NMR spectra. The blue arrows indicate the transfer steps that yield the peaks in the HSQC spectra, while the red arrows show the additional magnetization transfer during the TOCSY that result in the additional peaks found in the HSQC-TOCSY spectra. **e.** 2D 1H,31P-HSQC-TOCSY spectra of AMP (*top, blue*) and AMP-nsp9 (*bottom, red*). **f.** 2D 1H,1H-HSQC-TOCSY spectra of AMP-nsp9. Results shown in **b** and **c** are representative of at least 3 independent experiments.

The NiRAN domain has been shown to transfer nucleotide monophosphates (NMPs) from nucleotide triphosphates (NTPs) (referred to as NMPylation) to protein substrates, including nsp9^16^ and the nsp12 co-factors, nsp7^22^ and nsp8^23^. We observed NiRAN-dependent NMPylation of native nsp9, but not native nsp7 or nsp8 (Fig. 1b, Extended Data Fig. 3). Quantification of ^32^P incorporation and intact mass analyses suggests stoichiometric incorporation of NMPs into nsp9 (Extended Data Fig. 3g-j). Mutation of nsp9 Asn1 to Ala or Asp reduced NMPylation of nsp9 (Fig. 1c, Extended Data Fig. 4a), consistent with previous work that suggested NMPylation occurs on the backbone nitrogen of nsp9 Asn1 ^16^. To provide direct evidence that the amino terminus of nsp9 is NMPylated by the NiRAN domain, we performed nuclear magnetic resonance (NMR) spectroscopy of AMPylated nsp9. The 2D ^1^H,^31^P HSQC and 2D HSQC-TOCSY spectra confirm that the AMP is attached to the nitrogen backbone atom of Asn1 via a phosphoramidate linkage (Fig. 1d-f, Extended Data 4b, c).

## The NiRAN domain RNAylates nsp9

**Figure 2.**
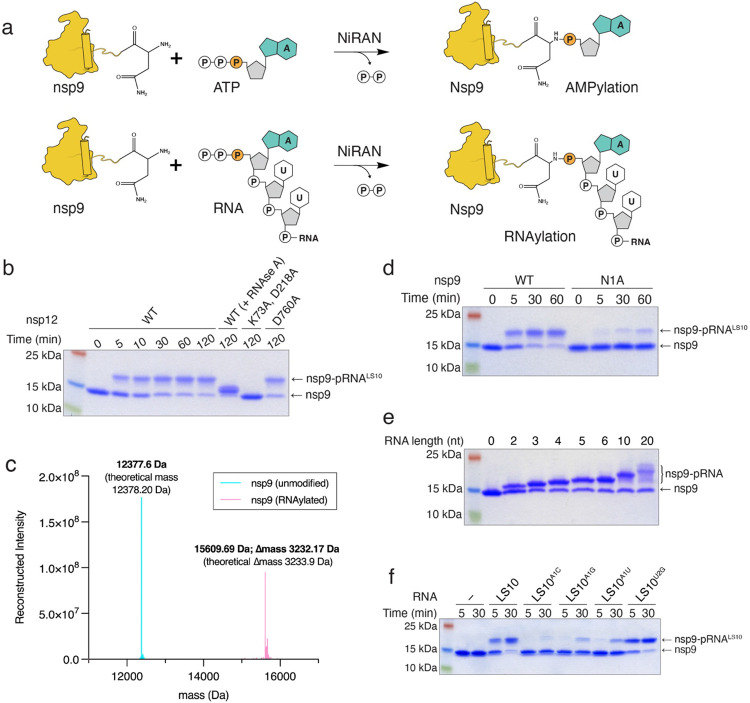
The NiRAN domain RNAylates nsp9. **a.** Schematic depicting the nsp9 AMPylation reaction (*top*) and the proposed nsp9 RNAylation reaction (*bottom*). **b.** Time-dependent incorporation of RNA into nsp9 by WT nsp12, the NiRAN mutant (K73A, D218A), or the polymerase mutant (D760A). Reaction products were analysed by SDS-PAGE and Coomassie staining. Samples were also treated with RNAse A. **c.** Intact mass LC/MS spectra (overlayed) of unmodified nsp9 (*cyan)* or nsp9 after incubation with 5′-pppRNA^LS10^ and WT nsp12 (*pink*). The theoretical and observed masses are shown in the insets. The Δmass of 3233.17 Da corresponds to monophosphorylated RNA^LS10^ (5′-pRNA^LS10^). **d.** Time-dependent incorporation of 5′-pRNA^LS10^ into nsp9 or the nsp9 N1A mutant. Reaction products were analysed as in **b**. **e.** Incorporation of different lengths of 5′-pppRNAs into nsp9 by the NiRAN domain. Reaction products were analysed as in **b**. **f.** Time-dependent incorporation of RNAs with substitutions in the first and second base into nsp9 by the NiRAN domain. Reaction products were analysed as in **b**. Results shown are representative of at least 2 independent experiments.

Given the ability of the NiRAN domain to transfer NMPs to nsp9 using NTPs as substrates, we wondered whether the NiRAN domain could also utilize 5′-pppRNA in a similar fashion (Fig. 2a). We synthesized a 5′-pppRNA 10-mer corresponding to the first 10 bases in the leader sequence (LS10) of the SARS-CoV-2 genome (hereafter referred to as 5′-pppRNA^LS10^) (Extended Data Table 1). We incubated 5′-pppRNA^LS10^ with nsp9 and nsp12 and analysed the reaction products by SDS-PAGE. Remarkably, we observed an electrophoretic mobility shift in nsp9 that was time-dependent, sensitive to RNAse A treatment and required an active NiRAN domain, but not an active RdRp domain (Fig. 2b). Intact mass analyses of the reaction products confirmed the incorporation of monophosphorylated RNA^LS10^ (5′-pRNA^LS10^) into nsp9 (Fig. 2c). The reaction was dependent on Mn^2+^ (Extended Data Fig. 5a) and required a triphosphate at the 5′-end of the RNA (Extended Data Fig. 5b). Substituting Ala for Asn1 reduced the incorporation of RNA^LS10^ into nsp9 (Fig. 2d). We also observed NiRAN-dependent RNAylation of nsp9 using LS RNAs ranging from 2 to 20 nucleotides (Fig. 2e). Mutation of the first A to any other nucleotide markedly reduced RNAylation (Fig. 2f). Thus, the NiRAN domain RNAylates the N-terminus of nsp9 in a substrate-selective manner.

## The NiRAN domain transfers 5′-pRNA from nsp9 to GDP forming the cap core structure GpppA-RNA

**Figure 3.**
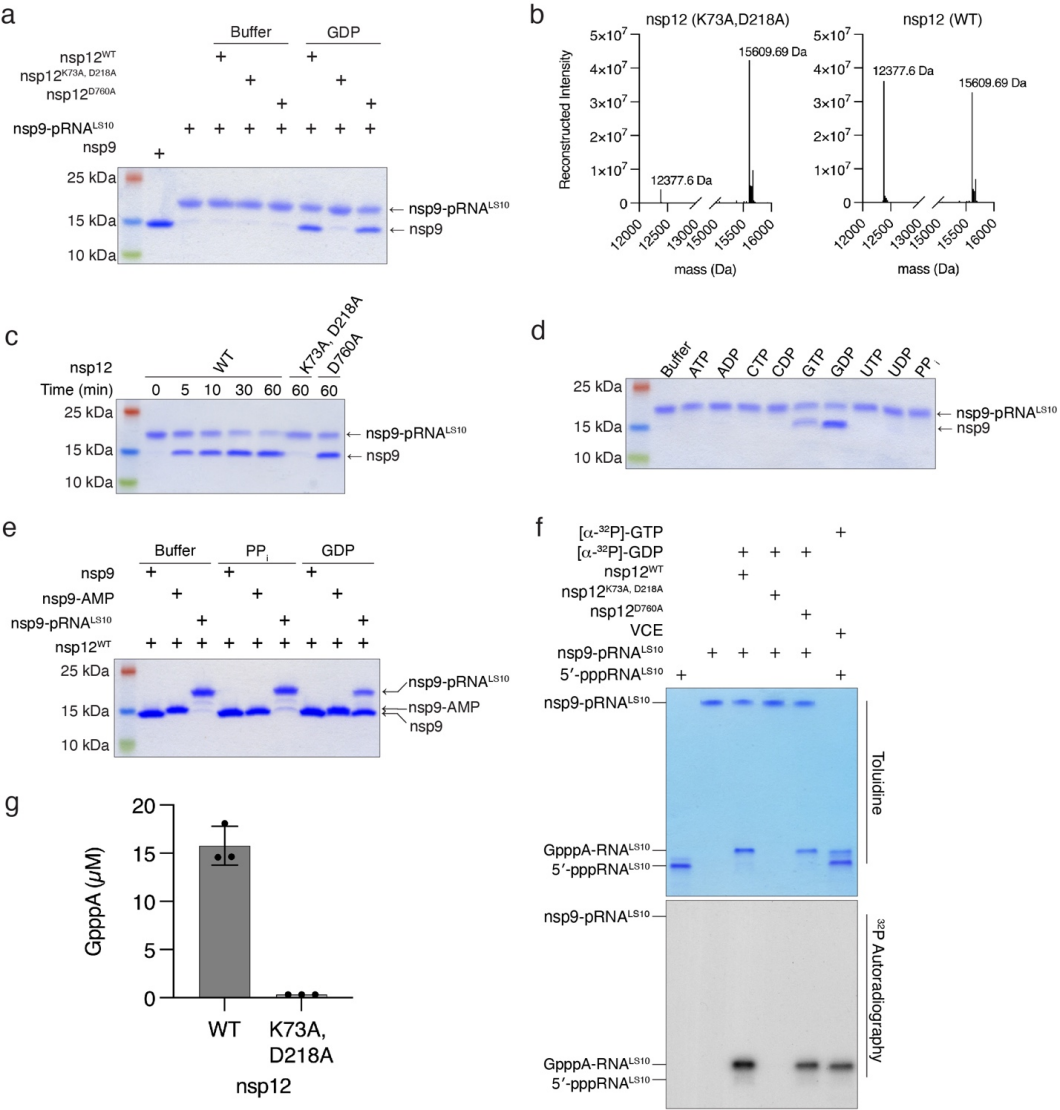
The NiRAN domain catalyses the transfer of 5′-pRNA from nsp9 to GDP to form the cap core structure GpppA-RNA. **a.** DeRNAylation of the covalent nsp9-RNA^LS10^ species by WT nsp12, the NiRAN mutant (K73A, D218A), or the polymerase mutant (D760A) when incubated with buffer or GDP. Reaction products were analysed as in Fig. 2b. **b.** Intact mass LC/MS spectra of nsp9-pRNA^LS10^ after incubation with GDP and WT nsp12 (*right)* or the NiRAN mutant (*left)*. The theoretical mass of nsp9 is 12378.2 Da and the theoretical mass of nsp9-pRNA^LS10^ is 15611.5 Da. **c.** Time-dependent deRNAylation of nsp9-pRNA^LS10^ by WT nsp12, the NiRAN mutant (K73A, D218A), or the polymerase mutant (D760A). Reaction products were analysed as in Fig. 2b. **d.** DeRNAylation of nsp9-pRNA^LS10^ by nsp12 in the presence of different NTPs, NDPs or PP_i_. Reaction products were analysed as in Fig. 2b. **e.** NiRAN-dependent deAMPylation or deRNAylation of nsp9 in the presence of PP_i_ or GDP. Reaction products were analysed as in Fig. 2b. **f.** Incorporation of α-^32^P from [α-^32^P]GDP into nsp9-pRNA^LS10^ by WT nsp12, the NiRAN mutant (K73A, D218A), or the polymerase mutant (D760A). Vaccinia capping enzyme (VCE) was used as a control but incubated with [α-^32^P]GTP. Reaction products were resolved by Urea-PAGE and visualized by toluidine blue O staining (upper) and autoradiography (lower). **g.** HPLC/MS quantification of GpppA formed during the NiRAN-catalysed deRNAylation of nsp9-pRNA^LS10^. Reaction products were digested with nuclease P1 prior to HPLC analysis. Reactions were performed in triplicate and error bars represent the standard deviation. Results shown are representative of at least 2 independent experiments.

Negative-sense RNA viruses of the order *Mononegavirales*, including vesicular stomatitis virus (VSV), have an unconventional capping mechanism in which a polyribonucleotidyltransferase (PRNTase) transfers 5′-pRNA from 5′-pppRNA to GDP via a covalent enzyme-RNA intermediate (Extended Data Fig. 6a)^24,25^. Because the NiRAN domain transfers 5′-pRNA to nsp9, we hypothesized that this protein-RNA species may be an intermediate in a similar reaction mechanism to that of the VSV system. To test this hypothesis, we purified the nsp9-pRNA^LS10^ species by ion exchange and gel filtration chromatography and incubated it with GDP in the presence of nsp12. Treatment with GDP deRNAylated nsp9 in a NiRAN-dependent manner, as judged by the nsp9 electrophoretic mobility on SDS-PAGE (Fig. 3a) and its molecular weight based on intact mass analysis (Fig. 3b). The reaction was time-dependent, (Fig 3c), preferred Mg^2+^ over Mn^2+^ (Extended Data Fig. 6b) and was specific for GDP—and to some extent GTP—but not the other nucleotides tested (Fig. 3d). Interestingly, although inorganic pyrophosphate (PP_i_) was able to deAMPylate nsp9-AMP, it was unable to deRNAylate nsp9-pRNA^LS10^ (Fig. 3e). (See Discussion)

We used Urea-PAGE to analyse the fate of the RNA^LS10^ during the deRNAylation reaction. Treatment of nsp9-pRNA^LS10^ with nsp12 and [α-^32^P]GDP resulted in a [^32^P]-labelled RNA species that migrated similarly to GpppA-RNA^LS10^ produced by the Vaccinia capping enzyme (Fig. 3f). The reaction was dependent on a functional NiRAN domain but not an active RdRp domain. To confirm the presence of a GpppA-RNA cap, we digested the RNA produced from the nsp12 reaction with P1 nuclease and detected GpppA by high performance liquid chromatography/mass spectrometry (HPLC/MS) analysis (Fig. 3g). Thus, the NiRAN domain is a GDP polyribonucleotidyltransferase (GDP-PRNTase) that mediates the transfer of 5′-pRNA from nsp9 to GDP.

In our attempts to generate GpppA-RNA^LS10^ in a “one pot” reaction, we found that GDP inhibited the RNAylation reaction (Extended Data Fig. 6c). However, the formation of GpppA-RNA^LS10^ could be generated in one pot provided that the RNAylation occurs prior to the addition of GDP (Extended Data Fig. 6c, d).

## Nsp14 and nsp16 catalyse the formation of the cap-0 and cap-1 structures

**Figure 4.**
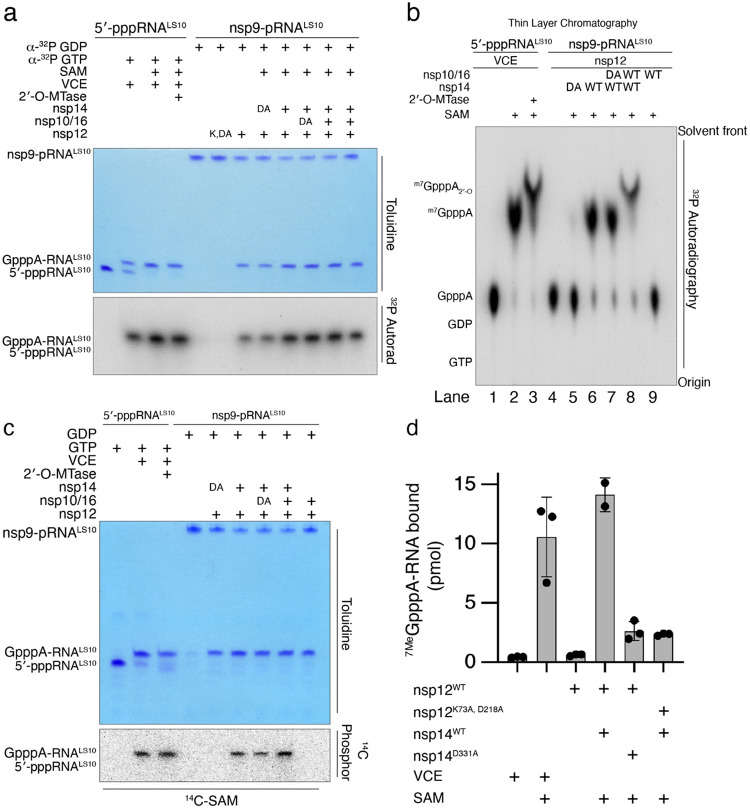
Nsp14 and nsp16 catalyse the formation of the cap-0 and cap-1 structures. **a.** Incorporation of α-^32^P from [α-^32^P]GDP into nsp9-pRNA^LS10^ by WT nsp12, or the NiRAN K73A, D218A mutant (K, DA). Reactions were subsequently incubated with SAM, nsp14 (or the D331A mutant; DA), and nsp10/16 (or the D130A nsp16 mutant; DA). The Vaccinia capping enzyme (VCE) was used as a positive control but incubated with [α-^32^P]GTP and the Vaccinia 2′-O-MTase. Reaction products were analysed as in Fig 3f. **b.** Thin layer chromatograms depicting the reaction products from Fig. 4a following extraction from the Urea PAGE gel and treatment with PI nuclease and CIP. Location of the cold standards (left) was visualized by UV fluorescence and the ^32^P by autoradiography. **c.** Incorporation of ^14^C from [methyl-^14^C]SAM into GpppA-RNA^LS10^ by nsp14 (or the D331A mutant; DA), and nsp10/16 (or the D130A nsp16 mutant; DA). The VCE and the Vaccinia 2′-O-MTase were used as positive controls. Reaction products were analysed as in Fig 3f. The ^14^C signal was detected by phophorimaging. **d.** Pull-down assays depicting the binding of [^32^P]^7Me^GpppA-RNA^LS10^ to GST-eIF4E. [^32^P]^7Me^GpppA-RNA^LS10^ was produced using SARS-CoV-2 virally encoded proteins or the VCE. Radioactivity in GST pull-downs was quantified by scintillation counting. Results represent three independent experiments. Error bars represent the standard deviation (SD). Results shown are representative of at least 2 independent experiments.

The SARS-CoV-2 genome encodes an N7-MTase domain within nsp14^6^ and a 2′-O-MTase in nsp16, the latter of which requires nsp10 for activity^7^. Nsp14 and the nsp10/16 complex use S-adenosyl methionine (SAM) as the methyl donor. To test whether NiRAN-synthesized GpppA-RNA^LS10^ can be methylated, we incubated ^32^P-labelled GpppA-RNA^LS10^ with nsp14 and/or the nsp10/16 complex in the presence of SAM and separated the reaction products by Urea-PAGE (Fig. 4a). We extracted RNA from the reaction, treated it with P1 nuclease and CIP, and then analysed the products by thin layer chromatography (TLC) (Fig. 4b). As expected, the NiRAN-synthesized cap migrated similarly to the GpppA standard and the products from the Vaccinia capping enzyme reaction (compare lanes 1 and 4). Likewise, reactions that included SAM and nsp14 migrated similarly to the ^7Me^GpppA standard and to the products from the Vaccinia capping enzyme reaction following the addition of SAM (compare lanes 2 and 6). Furthermore, treatment of ^7Me^GpppA-RNA^LS10^, but not unmethylated GpppA-RNA^LS10^, with nsp10/16 produced the ^7Me^GpppA_2′-O-Me_-RNA cap-1 structure (compare lanes 3, 8 and 9). In parallel experiments, we incubated NiRAN-synthesized GpppA-RNA^LS10^ with nsp14 and/or the nsp10/16 complex in the presence of [^14^C]-labelled SAM (^14^C on the donor methyl group) and separated the reaction products by Urea-PAGE. As expected, nsp14 and the nsp10/16 complex incorporated ^14^C into GpppA-RNA^LS10^ to form the cap-0 and cap-1 structures, respectively (Fig 4c). Thus, the SARS-CoV-2 ^7Me^GpppA_2′-O-Me_-RNA capping mechanism can be reconstituted in vitro using virally encoded proteins.

Efficient translation of mRNAs is dependent on eIF4E binding to the ^7Me^GpppA-RNA cap^26^. To test whether the SARS-CoV-2 RNA cap is functional, we incubated [^32^P]-labelled ^7Me^GpppA-RNA^LS10^ with GST-tagged eIF4E. We observed [^32^P]-labelled RNA in GST pulldowns of [^32^P]^7Me^GpppA-RNA but not the unmethylated derivative (Fig. 4d). Thus, the ^7Me^GpppA-RNA cap generated by SARS-CoV-2 encoded proteins is a substrate for eIF4E in vitro, suggesting that the cap is functional.

## Structural insights into RNA capping by the NiRAN domain

**Figure 5.**
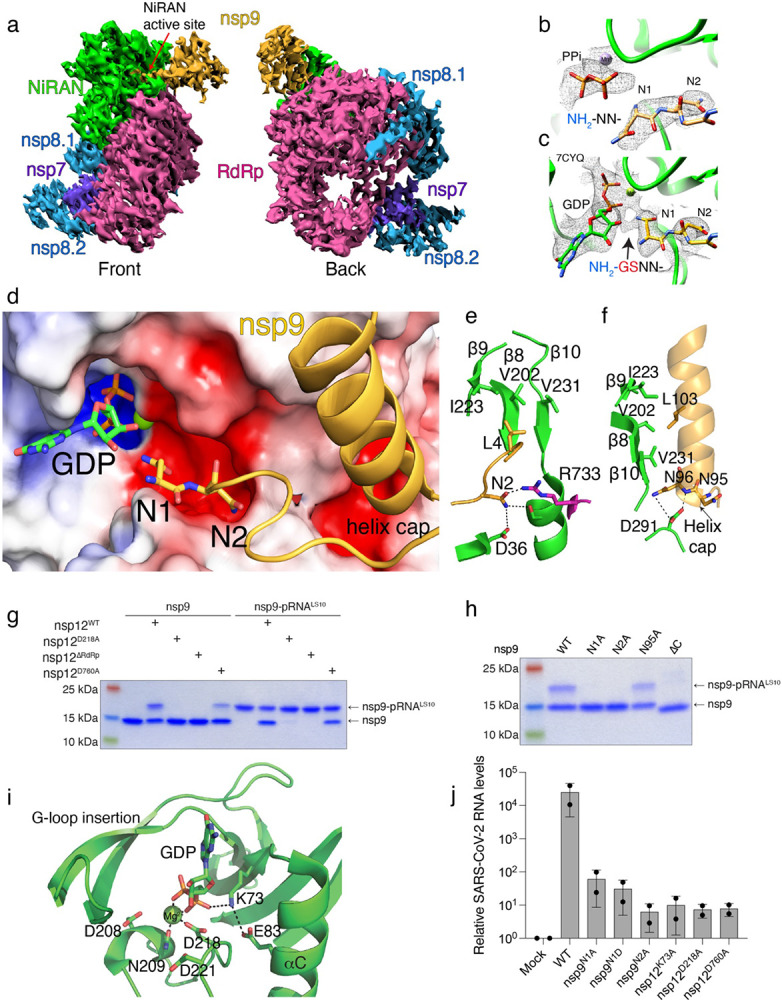
Structural and genetic insights into RNA capping by the kinase domain. **a.** Front and back views of nsp12/7/8/9 cryo-EM maps, with respect to the NiRAN domain. The NiRAN domain is in green, the RdRp in magenta, nsp7 in violet, nsp8 in light blue and nsp9 in gold. **b, c.** Coulomb density maps of the N-terminus of nsp9 from this study (**b)** and by Yan et al. (**c**) ^21^ (PDB ID:7CYQ). The NiRAN domain is shown in green and nsp9 is in gold. The arrow in (**c**) indicates additional density that likely corresponds to unmodeled Gly-Ser residues at the non-native N-terminus of nsp9. **d.** Electrostatic surface view of the NiRAN active site from 7CYQ bound to nsp9 (gold). The N-terminus and the C-terminal helix of nsp9 are shown. Electrostatic surface of nsp12 is contoured at 5 kT. **e.** Cartoon representation depicting the interactions between the nsp9 N-terminus (gold) with the β8-β9-β10 sheet in the NiRAN domain (green). Asn2 in nsp9 forms electrostatic interactions with Asp36 in the NiRAN domain and Arg733 in the RdRp domain (magenta). PDB ID 7CYQ was used. **f.** Cartoon representation depicting the interactions between the nsp9 C-terminal helix (gold) and the β8-β9-β10 sheet in the NiRAN domain (green). Interactions between Asn95/96 in nsp9 and D291 in the NiRAN domain are indicated. PDB ID 7CYQ was used. **g.** Incorporation of 5′-pRNA^LS10^ into nsp9 and deRNAylation of nsp9-pRNA^LS10^ by WT nsp12, the NiRAN mutant (D218A), the polymerase mutant (D760A), or the isolated NiRAN domain (residues 1-326; ΔRdRP). Reaction products were analysed as in Fig. 2b. **h.** Incorporation of 5′-pRNA^LS10^ into nsp9 (or the indicated mutants) by nsp12. Reaction products were analysed as in Fig. 2b. **i.** Cartoon representation of the NiRAN active site. Catalytic residues and GDP are shown as sticks, Mg^2+^ is a green sphere, and interactions are denoted by dashed lines. **j.** Relative viral yields from WT or mutant SARS-CoV-2 viruses bearing indicated mutations in nsp9 and nsp12. Data represent averages of two biological replicates. Error bars, SD. Results shown in **g** and **h** are representative of at least 2 independent experiments.

We determined a cryo-EM structure of the nsp7/8/9/12 complex and observed a nsp9 monomer bound in the NiRAN active site (Fig. 5a, Extended Data Fig. 7-9, Extended Data Table 2). The native N-terminus of nsp9 occupies a similar position to previously reported structures using a non-native N-terminus of nsp9 (Fig. 5b, c)^21^. Our cryo-EM analysis was hindered by the preferred orientation of the complex and sample heterogeneity, yielding final maps with high levels of anisotropy, with distal portions of nsp9 missing, and weak density for the N-lobe of the NiRAN domain (Extended Data Fig. 7, 8). Therefore, we used our model and the complex structure by Yan et al.^21^ (PDBID: 7CYQ) to study the structural basis of NiRAN-mediated RNA capping.

The first four residues of nsp9 extend into the NiRAN active site, forming electrostatic and hydrophobic contacts in and around a groove near the kinase-like active site (Fig. 5d). Asn1 of nsp9 is positioned inside of the active site, primed for transfer of 5′-pppRNA onto its N-terminus. Although the terminal NH_2_ group of nsp9 is the substrate for RNAylation, the local quality of the structures is not high enough to distinguish its exact position. We have modelled the nsp9 acceptor NH_2_ pointing towards what appears to be the phosphates of the nucleotide analogue UMP-NPP in the active site (Fig 5b). In the structure by Yan et al.^21^, Asn1 was assigned an opposite conformation and there are unmodeled residues (non-native N-terminus; NH_2_-Gly-Ser-) visible in the density maps, distorting local structural features (Fig. 5c, arrow)^27^.

Asn2 of nsp9 is in a negatively charged cleft around the NiRAN active site, and contacts Arg733, which extends from the polymerase domain and is partially responsible for positioning nsp9 (Fig. 5e). Both Leu4 and the C-terminal helix of nsp9 form hydrophobic interactions with a β-sheet (β8-β9-β10) in the N-lobe of the NiRAN domain (Fig. 5e, f). The N-terminal cap of the nsp9 C-terminal helix also forms electrostatic interactions with a negatively charged pocket on the surface of the NiRAN domain (Fig. 5f). Nsp12 lacking the RdRp domain (ΔRdRp; 1-326) neither RNAylates nsp9 nor processes nsp9-pRNA^LS10^ to form GpppA-RNA (Fig. 5g). Likewise, deleting the C-terminal helix on nsp9 (ΔC; 1-92) and Ala substitutions of Asn1 and Asn2 abolished RNAylation (Fig. 5h).

**Extended Data Fig. 1.**
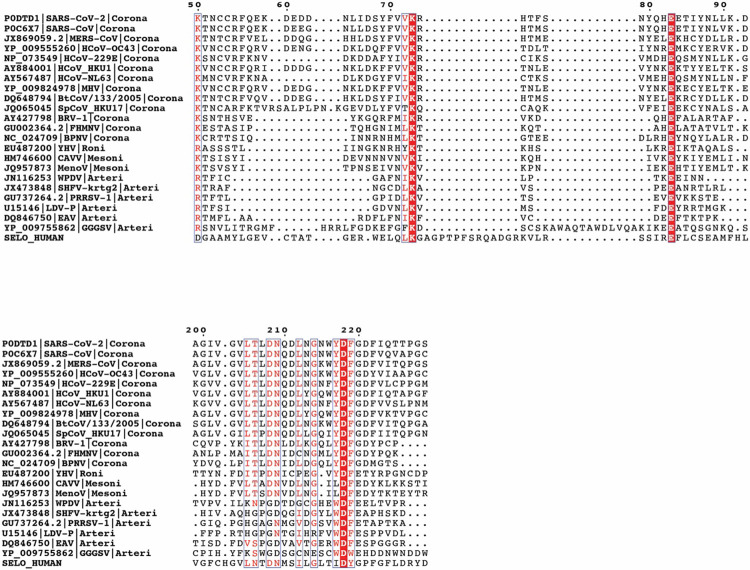
Sequence alignment of the NiRAN domain reveals similarity to the pseudokinase selenoprotein-O (SelO). Multiple sequence alignment highlighting conserved kinase-like active site residues in the NiRAN domain among several CoVs, other selected *Nidovirales* (Arteri-, Mesoni- and Roniviruses) and the human SelO pseudokinase. Top: amino acid sequence surrounding the Lys-Glu ion pair. Bottom: and the amino acid sequence surrounding the active site and the “DFG” motif.

The NiRAN domain resembles SelO, with an RMSD of 5.7 Å over 224 Cα atoms (PDB ID: 6EAC^13^, Extended Data Fig. 10a). Lys73 (PKA nomenclature; K72) forms a salt bridge with Glu83 (PKA; E91) from the αC (α2) helix and contacts the phosphates (GDP in 7CYQ, or UMP-NPP in our structure; Fig. 5i). As expected, the “DFG” Asp218 (PKA; D184) binds a divalent cation. Interestingly, the NiRAN domain lacks the catalytic Asp (Extended Data Fig. 1), (PKA; D166); however, like in SelO, Asp208 is next to the metal binding Asn209 (PKA; N171) and may act as a catalytic base to activate the NH_2_ group on the N-terminus of nsp9 (Fig. 5i).

In canonical kinases, the β1-β2 G-loop stabilizes the phosphates of ATP^28^. In contrast, the NiRAN domain contains a β-hairpin insert (β2-β3) where the β1-β2 G-loop should be (Extended Data Fig. 10b). This insertion not only makes contacts with the N-terminus of nsp9, but also contains a conserved Lys (K50) that extends into the active site and stabilizes the phosphates of the bound nucleotide. Likewise, Arg116 also contacts the phosphates of the nucleotide. SelO contains a similar set of basic residues pointing into the active site that accommodate the flipped orientation of the nucleotide to facilitate AMPylation (Extended Data Fig. 10b). Notably, Lys73, Arg116 and Asp218 in SARS-CoV-1 nsp12 are required for viral replication^5^.

## The kinase-like residues of the NiRAN domain and the N-terminus of nsp9 are essential for SARS-CoV-2 replication

To determine the importance of the NiRAN domain and the N-terminus of nsp9 in viral replication, we used a DNA-based reverse genetics system that can rescue infectious SARS-CoV-2 (Wuhan-Hu-1/2019 isolate) expressing a fluorescent reporter^8^ (Extended Data Fig. 11a). We introduced single point mutations in nsp9 (N1A, N1D and N2A) and nsp12 (K73A, D218A and D760A) and quantified the virus in supernatants of producer cells by RT-qPCR to detect the viral N gene. We observed a 400 to 4000-fold reduction in viral load for all the mutants compared to WT (Fig. 5j, Extended Data Fig. 11a). To account for the possibility of a proteolytic defect in the mutant viral polyprotein, we tested whether the main viral protease nsp5 (M^Pro^) can cleave a nsp8-nsp9 fusion protein containing the Asn1/Asn2 mutations in nsp9. The N1D mutant failed to be cleaved by nsp5, suggesting that the replication defect observed for this mutant is a result of inefficient processing of the viral polyprotein. However, the N1A and N2A mutants were efficiently cleaved by nsp5 (Extended Data Fig. 11b, c). Collectively, these data provide genetic evidence that the residues involved in capping of the SARS-CoV-2 genome are essential for viral replication.

## Discussion

**Figure 6.**
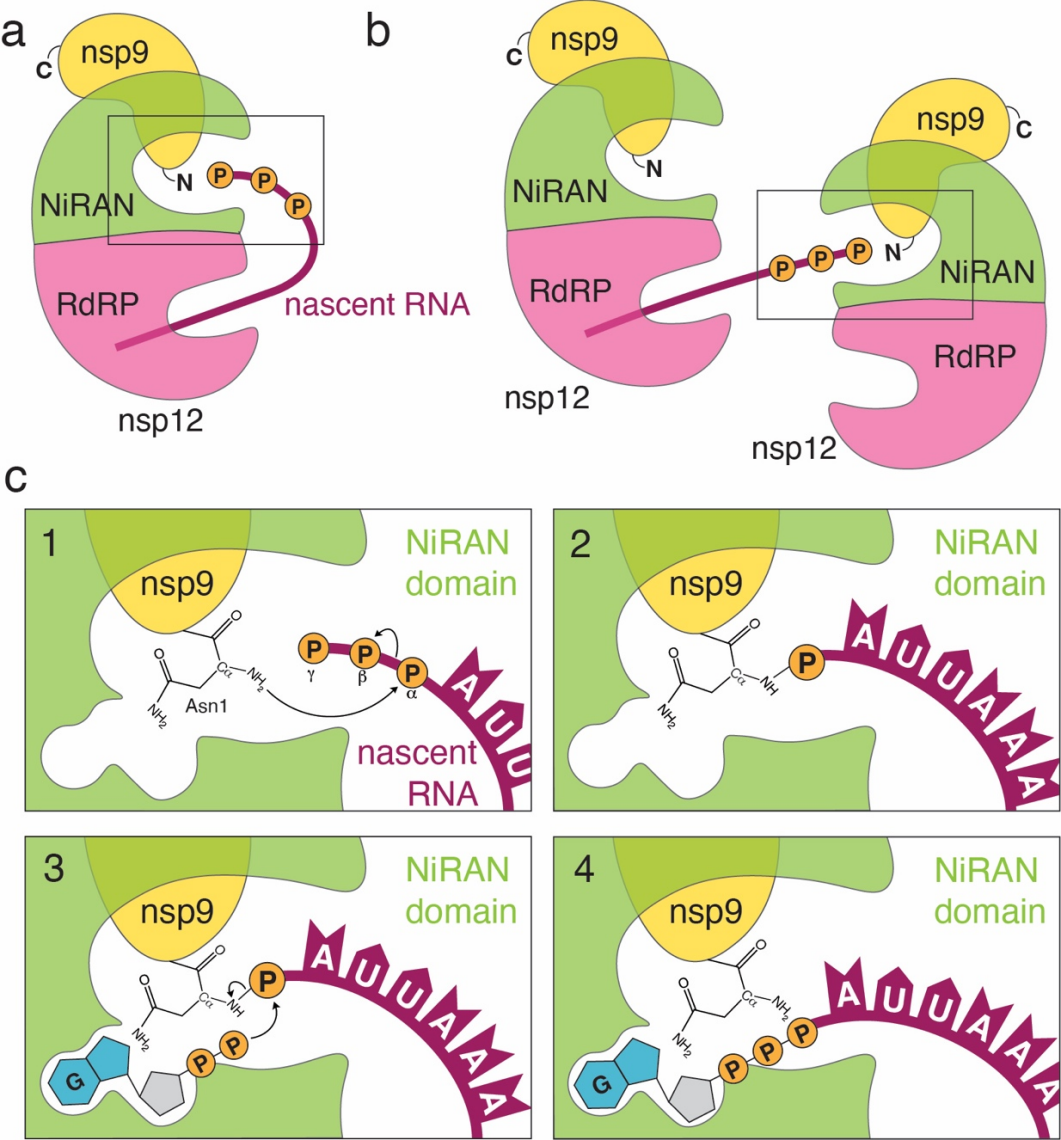
Proposed model of the SARS-CoV-2 RNA capping mechanism **a, b.** During transcription, the nascent 5′-pppRNA binds to the NiRAN active site in either a *cis* (**a**) or a *trans* (**b**) manner. **c.** Upon binding, the N-terminus of nsp9 attacks the α-phosphate of the nascent 5′-pppRNA (**1**), forming the covalent nsp9-pRNA species and releasing PP_i_ (**2**). Upon GDP binding to the NiRAN active site, the β-phosphate of the nsp13-generated GDP attacks the 5′-phosphate on the nsp9-pRNA (**3**), releasing capped RNA and regenerating unmodified nsp9 (**4**). Subsequent methylation events are carried out by nsp14 and nsp16 to generate the ^7Me^GpppA_2′-O-Me_-RNA cap.

We propose the following mechanism of RNA capping by CoV: during transcription, the nascent 5′-pppRNA binds to the NiRAN active site, in either a *cis* (Fig. 6a) or *trans* (Fig. 6b) manner and 5′-pRNA is subsequently transferred to the N-terminus of nsp9 forming a phosphoramidate bond (Fig. 6c, **panels 1 and 2)**. The nsp13 protein produces GDP from GTP, which binds the NiRAN active site and attacks RNAylated nsp9, releasing capped RNA and regenerating unmodified nsp9 (Fig. 6c, **panels 3 and 4)**. Subsequently, nsp14 and nsp16 perform sequential N7 and 2′-O methylations, forming a fully functional ^7Me^GpppA_2′-O-Me_-RNA cap.

SARS-CoV-2 nsp12 is thought to initiate transcription/replication starting with an NTP, or a short 5′-pppRNA primer^29^. Cryo-EM structures of the RTC suggest that the dsRNA product makes its way out of the RdRp active site in a straight line, supported by the nsp8 helical stalks^21,30,31^. In a *cis* capping model, the helical duplex with nascent 5′-pppRNA would then need to unwind, flex 90°, and extend into the NiRAN active site ~70 Å away (Fig. 6a). More likely, a separate RTC complex could perform capping in *trans* (Fig. 6b). Notably, Perry et al.^32^ propose that the nascent RNA strand is separated from the template upon passing through the proof-reading ExoN domain of nsp14 on a neighbouring RTC and threaded towards the NiRAN domain.

SARS-CoV-1 nsp13 has RNA helicase, nucleotide triphosphatase (NTPase), and RNA 5′-triphosphatase (RTPase) activities^20^. The RTPase activity implicated nsp13 in the first step of the capping mechanism; however, while nsp13 can act on 5′-pppRNA, this reaction is inhibited in the presence of cellular concentrations of ATP^20^. Thus, we favour the idea that the physiological functions for nsp13 are: **1)** to utilize the energy from ATP hydrolysis to unwind double-stranded RNA (helicase), and **2)** to hydrolyse GTP to GDP, which can then act as an acceptor for 5′-pRNA in the NiRAN-catalysed capping reaction.

The SARS-CoV-2 capping mechanism is reminiscent of the capping mechanism used by VSV, although there are some differences. The VSV large (L) protein is a multifunctional enzyme that carries out RdRp, PRNTase, and methyltransferase activities to form the cap^24,33,34^. During the reaction, 5′-pRNA is transferred to a conserved His within the PRNTase domain, which adopts a unique α-helical fold that is distinct from that of protein kinases^25^. The presence of two different enzymatic mechanisms of capping, proceeding via covalent protein-RNA intermediates, in *Mononegavirales* and in *Nidovirales* is an example of convergent evolution.

Consistent with other reports^16,27^, we observed NiRAN-catalysed NMPylation of nsp9 (Fig. 1b, Extended Data Fig. 3). While our results do not necessarily preclude a biologically relevant function for nsp9 NMPylation, it is worth noting that this modification is reversible in the presence of PP_i_^27^ (Fig. 3e). PP_i_ is produced during the RdRp reaction, making the stability of NMPylated nsp9 difficult to envision in vivo. By contrast, RNAylated nsp9 was not reversible in the presence of PP_i_. Thus, RNAylation is likely the physiologically relevant modification of nsp9 during viral RNA capping.

Recent work suggested that the NiRAN domain is a GTase that transfers GMP from GTP to 5′-ppRNA, forming a GpppA-RNA cap intermediate^3,21^. In our efforts to reproduce these results, we failed to detect nsp12-dependent GpppA cap formation by TLC (Extended Data Fig. 12a) or by Urea-PAGE analysis of the RNA (Extended Data Fig. 12b), in contrast to our control, in which the Vaccinia capping enzyme efficiently generated GpppA-RNA. Because nsp13 and the Vaccinia capping enzyme can hydrolyse GTP to GDP^20^, the cap reported previously^21^ appears to be GDP formed from nsp13- and Vaccinia capping enzyme-dependent hydrolysis of GTP.

In summary, we have defined the mechanism by which SARS-CoV-2 caps its genome and have reconstituted this reaction in vitro using non-structural proteins encoded by SARS-CoV-2. Our results uncover new targets for the development of antivirals to treat COVID-19 and highlight the catalytic adaptability of the kinase domain.

## Methods

### Chemicals and reagents

Ampicillin sodium (A9518), ATP (A2383), ADP (A2754), chloramphenicol (C0378), CTP (C1506), CDP (C9755), dithiothreitol (DTT; D0632), EDTA (E5134), GTP (G8877), GDP (G7127), imidazole (I2399), IPTG (I5502), kanamycin sulfate (K1377), 2-mercaptoethanol (BME, M3148), Brilliant blue R (B0149), magnesium chloride (MgCl_2_; M2670), manganese (II) chloride tetrahydrate (MnCl_2_; M3634), PEI-cellulose TLC plates (Z122882), potassium chloride (KCl; P9541), pyrophosphate (221368), Urea (U6504), UTP (U6625), UDP (94330), were obtained from MilliporeSigma (St. Louis, MO). Q5 DNA polymerase (M0492L), all restriction enzymes used for cloning, Proteinase K (P8107S), Yeast Inorganic Pyrophosphatase (M2403), Quick CIP (M0525S), Nuclease P1 (M0660S), Vaccinia Capping System (M2080S), mRNA Cap 2'-O-Methyltransferase (M0366S), G(5′)ppp(5′)A RNA Cap Structure Analog (GpppA; S1406L), and m7G(5′)ppp(5′)A RNA Cap Structure Analog (m7GpppA; S1405S) were all obtained from New England Biolabs (Ipswich, MA). Acetic acid (A38-212), RNAse inhibitor (N8080119), 2X TBE-Urea Sample Buffer (LC6876), and isopropanol (42383) were all obtained from Thermo Fisher Scientific (Waltham, MA). [α-^32^P]-ATP (BLU003H250UC), [α-^32^P]-CTP (BLU008H250UC), [α-^32^P]-GTP (BLU006H250UC), [α-^32^P]-UTP (BLU007H250UC), and S-[methyl-^14^C]-Adenosyl-L-Methionine (NEC363010UC) were all obtained from PerkinElmer (Waltham, MA). All 5′-triphosphorylated RNAs were custom synthesized by ChemGenes Corporation (Wilmington, MA). Phenylmethylsulfonyl fluoride (PMSF; 97064-898) was obtained from VWR (Radnor, PA).

4–20% Mini-PROTEAN® TGX Stain-Free™ Protein Gels (4568096) were obtained from Bio-Rad. Uridine-5′-[(α,β)-imido]triphosphate (UMP-NPP; NU-930L) was obtained from Sapphire North America (Ann Arbor, MI).

### Plasmids

SARS-CoV-2 nsp7, nsp8, nsp12, nsp13, nsp14, and nsp16 coding sequences (CDS) were codon-optimized for bacterial expression and synthesized as gBlocks (Integrative DNA Technologies, Coralville, IA). The CDS for nsp9 and nsp10 were amplified from mammalian expression vectors (a generous gift Nevan Krogen) ^35^. The CDS were cloned into modified pET28a bacterial expression vectors containing N-terminal 6/8/10xHis tags followed by the yeast Sumo (smt3) CDS Amino acid mutations were introduced via QuikChange site-directed mutagenesis. Briefly, primers were designed using the Agilent QuikChange primer design program to generate the desired mutation and used in PCR reactions with PfuTurbo DNA polymerase. Reaction products were digested with Dpn1, transformed in DH5α cells and mutations were confirmed by Sanger sequencing.

For protein expression in *Escherichia coli* (*E. coli*), ppSumo-SARS-CoV-2 nsps and mutants were cloned into a BamH1 site at the 5′ end, which introduced a Ser residue following the diGly motif in smt3. To make native N-termini, the codon encoding the Ser was deleted via QuickChange mutagenesis. Thus, following cleavage with the ULP protease (after the diGly motif), the proteins contained native N-termini.

pGEX-2T-GST-eIF4E K119A ^36^ was obtained from Addgene (plasmid # 112818 )

### Protein purification

#### Nsp5. nsp7, nsp8, nsp10, nsp14 and nsp16

6xHis-Sumo-nsp5/7/8/10/14/16 and corresponding mutant plasmids (with native N-termini following the diGly motif in Sumo) were transformed into Rosetta (DE3) *E. coli* or LOBSTR-BL21(DE3)-RIL cells under 50 μg/ml kanamycin exposure. 5 ml LB Miller growth medium starter cultures containing 50 μg/ml kanamycin and 34 μg/ml chloramphenicol were grown for 2-4 h at 37°C and then transferred to growth medium containing the same antibiotics. Typically, 2 L were grown for each protein. Protein expression was induced at O.D. 0.7-1.0 by adding 0.4 mM IPTG and overnight incubation (16 hours) at 18°C. Cultures were centrifuged at 3,000 x g for 10 min and the bacterial pellet was resuspended in lysis buffer (50 mM Tris pH 8.0, 300 mM NaCl, 17.4 μg/ml PMSF, 15 mM imidazole pH 8.0 and 5 mM β-ME) and lysed by sonication. Lysates were centrifuged for 30 min at 30,000-35,000 x g and the supernatants incubated with Ni-NTA resin for 1-2 h at 4°C. The Ni-NTA resin was washed with 50 mM Tris pH 8.0, 300 mM NaCl, 30 mM imidazole pH 8.0,1 mM DTT and the protein was eluted in 50 mM Tris pH 8.0, 300 mM NaCl, 300 mM imidazole pH 8.0 and 1 mM DTT. The eluted proteins were incubated with 5 μg/ml Ulp1 protease overnight at 4°C. Nsp7 was separated from 6xHis-Sumo by anion exchange (Capto HiRes Q 5/50 column (Cytiva) equilibrated in 50 mM Tris 8.0, 50 mM NaCl, 1 mM DTT, eluted with 0-50% gradient of buffer containing 1 M NaCl). 6xHis-SUMO-nsp8 was treated with Ulp1 on the Ni-NTA resin, to separate nsp8 and 6xHis-SUMO in buffer with no imidazole. Proteins were further purified by size exclusion chromatography using Superdex 200 10/300 increase, Superdex 200 16/600, Superdex 75 10/300 increase or Superdex 75 16/600 columns in 50 mM Tris pH 7.5-8.0, 150-300 mM NaCl, 1 mM DTT, depending on yield and size. Fractions containing proteins of interest were pooled, concentrated in an Amicon Utra-15 with a 3-50 kDa pore size centrifugal filters. The nsp16 protein was incubated with Ni-NTA resin post SEC to remove 6His-SUMO. Purified proteins were aliquoted and stored at −80°C.

#### Nsp9

6xHis-Sumo-nsp9 and respective mutant plasmids (with native N-termini following the diGly motif in Sumo) were transformed into Rosetta (DE3) *E. coli* cells under 50 μg/ml kanamycin exposure. 5 ml LB Miller growth medium starter cultures containing 50 μg/ml kanamycin and 34 μg/ml chloramphenicol were grown for 2-4 h at 37°C and then transferred to Terrific Broth (TB) growth medium containing the same antibiotics and several drops of Antifoam B emulsion (Sigma, A5757). Protein expression was induced at O.D 1.2 by adding 0.4 mM IPTG and overnight incubation (16 hours) at 18°C. Cultures were centrifuged at 3,000 x g for 10 min and the bacterial pellet was resuspended in lysis buffer (50 mM Tris pH 8.0, 300 mM NaCl, 10% glycerol 17.4 μg/ml PMSF, 15 mM imidazole pH 8.0 and 5 mM β-ME) and lysed by sonication. Lysates were centrifuged for 30 min at 30,000 x g and the supernatants incubated in Ni-NTA resin for 1-2 h at 4°C. The Ni-NTA resin was washed with 50 mM Tris pH 8.0, 300 mM NaCl, 10% glycerol, 30 mM imidazole pH 8.0, 1 mM DTT and each protein was eluted in 50 mM Tris pH 8.0, 300 mM NaCl, 10% glycerol, 300 mM imidazole pH 8.0 and 1 mM DTT. The eluted protein was incubated with 5 μg/ml Ulp1 overnight at 4°C. Proteins were diluted with 50 mM Tris pH 8.0, 10% glycerol, 1 mM DTT buffer to lower the NaCl concentration to 30 mM and subsequently ran through a Hi-Trap CaptoQ column where the flowthrough contained purified nsp9. NaCl was added to each protein to a final concentration of 150 mM, concentrated in an Amicon Ultra-15 with a 10k MWCO, aliquoted, and stored at −80°C.

#### Nsp12

8xHis or 10xHis-Sumo-nsp12 and respective mutant plasmids (with native N-termini following the diGly motif in Sumo) were transformed into LOBSTR-BL21(DE3)-RIL *E. coli* cells under 50 μg/ml kanamycin exposure. 5 ml LB Miller growth medium starter cultures containing 50 μg/ml kanamycin and 34 μg/ml chloramphenicol were grown for 2-4 h at 37°C and then transferred to 1L growth medium containing the same antibiotics. Protein expression was induced at O.D. 0.8-1.2 by adding 0.4 mM IPTG and overnight incubation (16 hours) at 18°C. Cultures were centrifuged at 3,000-3.500 x g for 10 min and the bacterial pellet was resuspended in lysis buffer (50 mM Tris pH 8.0, 300 mM NaCl, 10% glycerol, 17.4 μg/ml PMSF, 15 mM imidazole pH 8.0 and 5 mM β-ME) and lysed by sonication. Lysates were centrifuged for 30 min at 30,000-35,000 x g and the supernatants incubated in Ni-NTA resin for 1-2 h at 4°C. The Ni-NTA resin was washed with high salt buffer: 50 mM Tris pH 8.0, 1 M NaCl, 10% glycerol, 30 mM Imidazole and 5 mM β-ME, followed by a high imidazole wash: 50 mM Tris pH 8.0, 300 mM NaCl, 10% glycerol, 75 mM Imidazole and 5 mM β-ME, and the protein was eluted in 50 mM Tris pH 8.0, 300 mM NaCl, 10% glycerol, 300 mM imidazole pH 8.0 and 1 mM DTT. The eluted proteins were incubated with 5 μg/ml Ulp1 overnight at 4°C. Proteins were further purified by size exclusion chromatography using a Superdex 200 10/300 increase column, or Superdex 200 16/600 in 50 mM Tris pH 8.0, 150-300 mM NaCl, 1 mM DTT. Fractions containing nsp12 were pooled, concentrated in an Amicon Ultra-15 with a 30-50k MWCO centrifugal filter, aliquoted, and stored at −80°C.

#### Nsp13

6xHis-Sumo-nsp13 (used in Extended Data Fig. 12), or 10xHis-Sumo-nsp13 (used for [α^32^P]-GTP conversion into GDP) and respective mutant plasmids (with native N-termini following the diGly motif in Sumo) were transformed into Rosetta (DE3) *E. coli* cells under 50 μg/ml kanamycin exposure. 5 ml LB Miller growth medium starter cultures containing 50 μg/ml kanamycin and 34 μg/ml chloramphenicol were grown for 2-4 h at 37°C and then transferred to 1 L of growth medium containing the same antibiotics. Protein expression was induced at O.D. 1.0 by adding 0.4 mM IPTG and overnight incubation (16 hours) at 18°C. Cultures were centrifuged at 3,000-3.500 x g for 10 min and the bacterial pellet was resuspended in lysis buffer (50 mM Tris pH 8.0, 300 mM NaCl, 10% glycerol, 17.4 μg/ml PMSF, 15 mM imidazole pH 8.0 and 5 mM β-ME) and lysed by sonication. Lysates were centrifuged for 30 min at 30,000-35,000 x g and the supernatants incubated in Ni-NTA resin for 1-2 h at 4°C. Ni-NTA resin for 6xHis-Sumo-nsp13 was washed with 50 mM Tris pH 8.0, 300 mM NaCl, 10% glycerol, 30 mM imidazole pH 8.0 and 1 mM DTT. 10xHis-Sumo-nsp13 with high salt buffer: 50 mM Tris pH 8.0, 1 M NaCl, 10% glycerol, 30 mM Imidazole and 5 mM β-ME, followed by a high imidazole wash: 50 mM Tris pH 8.0, 300 mM NaCl, 10% glycerol, 75 mM imidazole and 5 mM β-ME, and the protein was eluted in 50 mM Tris pH 8.0, 300 mM NaCl, 10% glycerol, 300 mM imidazole pH 8.0 and 1 mM DTT. The eluted protein was incubated with 5 μg/ml Ulp1 overnight at 4°C. Proteins were buffer-exchanged or dialysed into a buffer containing 50 mM Bis-Tris pH 6.0, 30 mM NaCl, 10% glycerol and 1 mM DTT followed by ion-exchange chromatography in a 5/50 MonoS column. Fractions containing nsp13 were pooled and further purified by size exclusion chromatography using a Superdex 200 10/300 increase or Superdex 200 16/600 column in 50 mM Tris pH 8.0, 150 mM NaCl, 10% glycerol (only in 6xHis), 1 mM DTT. Fractions containing nsp13 were pooled, concentrated in an Amicon Ultra-15 with a 30-50k MWCO, aliquoted, and stored at −80°C.

#### eIF4E

For production of GST-eIF4E K119A, LOBSTR-BL21(DE3)-RIL cells were transformed with pGEX-2T-GST-eIF4E K119A ^36^ and were grown in LB supplemented with 100 μg/L Ampicillin, 34 μg/L chloramphenicol. Protein expression was induced at O.D. 1.0 by adding 0.4 mM IPTG and overnight incubation (16 hours) at 18 °C. Cultures were centrifuged at 3,000-3.500 x g for 10 min and the bacterial pellet was resuspended in lysis buffer (50 mM Tris pH 8.0, 300 mM NaCl, 17.4 μg/ml PMSF, and 5 mM β-ME) and lysed by sonication. Lysates were centrifuged for 30 min at 35,000 x g and the supernatants incubated with Pierce Glutathione resin for 1-2 h at 4 °C. The resin was washed with lysis buffer, and the GST-eIF4E K119A eluted with 50 mM Tris-HCl pH 8.0, 300 mM NaCl, 50 mM glutathione, 1 mM DTT. The protein was purified over Size Exclusion Chromatography on Superdex 200 16/600 in 50mM Tris-HCl pH 8.0, 300 mM NaCl, 1 mM DTT, concentrated, and stored as above.

#### Ipp1

For production of yeast inorganic pyrophosphatase (ipp1), The S. cerevisiae ipp1 CDS was cloned into pProEx2 containing a N-terminal 6xHis-TEV linker and was transformed into Rosetta *E. coli* cells under 100 μg/ml ampicillin exposure. 5 ml LB Miller growth medium starter cultures containing 100 μg/ml ampicillin were grown for 2-4 h at 37°C and then transferred to 1L growth medium containing the same antibiotics. Protein expression was induced at O.D. 0.8-1.2 by adding 0.4 mM IPTG and overnight incubation (16 hours) at 18°C. Cultures were centrifuged at 3,000-3.500 x g for 10 min and the bacterial pellet was resuspended in lysis buffer (50 mM Tris pH 8.0, 300 mM NaCl, 17.4 μg/ml PMSF, 15 mM imidazole pH 8.0 and 5 mM β-ME) and lysed by sonication. Lysates were centrifuged for 30 min at 35,000 x g and the supernatants incubated in Ni-NTA resin for 1 h at 4°C. Ni-NTA resin was washed with high salt buffer: 50 mM Tris pH 8.0, 1 M NaCl, 30 mM imidazole and 5 mM β-ME, and was eluted in 50 mM Tris pH 8.0, 50 mM NaCl, 300 mM imidazole pH 8.0 and 1 mM DTT. The eluted protein was loaded onto a Capto HiRes Q 5/50 column (Cytiva) equilibrated in 50 mM Tris 8.0, 50 mM NaCl, 1 mM DTT, eluted with 0-50% gradient of buffer containing 1 M NaCl. Protein was further purified by size exclusion chromatography using a Superdex 200 16/600 in 25 mM Tris pH 7.5, 50 mM NaCl, 2 mM DTT. Fractions containing YIPP were pooled, concentrated, and stored as above.

#### Nsp8-nsp9 fusion

The 6xHis-Sumo-nsp8-nsp9 plasmid and mutants (N1A, N1D and N2A) were transformed into Rosetta (DE3) *E. coli*. Cells were grown in Terrific broth media in the presence of 50 μg/ml kanamycin and 25 μg/ml chloramphenicol to OD 1.0 and induced with 0.4 mM IPTG for 16 hours at 18 °C. Cultures were centrifuged at 3500 x g for 15 minutes, and the pellets resuspended in lysis buffer (50 mM Tris, pH 8.0; 500 mM NaCl; 25 mM imidazole; 10% glycerol) in the presence of 1 mM PMSF. Cells were lysed by sonication and lysates cleared by centrifugation at 25000 x g for 1 hour. The lysate was passed over Ni-NTA beads, which were washed with lysis buffer. Protein samples were eluted with elution buffer (50 mM Tris, pH 8.0; 300 mM NaCl; 300 mM imidazole; 5% glycerol) and cleaved overnight at 4 °C with Ulp Sumo protease. Protein samples were further purified into cleavage assay buffer (50 mM Tris, pH 7.4; 150 mM NaCl; 5% glycerol) by size exclusion chromatography using a Superdex 75 Increase 10/300 GL column.

### Guanylyltransferase (GTase) activity assays

GTase activity assays were performed as described in Yan et al. ^21^. Reactions were assembled in 20 μL containing 50 mM Tris pH 8.0, 5 mM KCl, 1 mM DTT, 0.005 U/ml inorganic pyrophosphatase, 10 uM 5′-pppACCCCCCCCCCCCCCCCCCC-3’ (5′-pppRNA^A19C^), 1.25 mM RNAse inhibitor, and where indicated, 0.5 μM nsp12, nsp12^D218A^, nsp13, nsp13^K288A^ or 1 U/ml of vaccinia capping enzyme (VCE). Reactions were started with 1 mM MgCl_2_, 100 μM [α-^32^P] GTP, (specific radioactivity = 1000 cpm/pmol) and incubated for 1 hr at 37°C. Half of the reaction was stopped by the addition of 0.8 U/ml proteinase K and incubated for 30 min at 37°C prior to the addition of 2X RNA loading dye (Novex) and incubated for 3 mins at 95°C. Reaction products were resolved in a 15% TBE-Urea PAGE gel. The gel was then stained with toluidine blue O and the ^32^P signal detected via autoradiography.

The other half of the GTase reactions were treated with 10 U/ml P1 nuclease for 1 h at 37°C. Reactions were then split in half again with one half treated with 1 U/ml Quick CIP for 30 min at 37°C. Reactions were spotted on a PEI cellulose thin-layer chromatography (TLC) plate and developed in a 0.4 M ammonium sulfate (NH_4_)_2_SO_4_ solvent system. The plate was dried and the ^32^P signal was detected via autoradiography.

### NMPylation assays

NMPylation reactions were carried out in 20 μL containing 50 mM Tris (pH 7.5), 5 mM KCl, 1 mM DTT, 16 μM nsp7, nsp8 or nsp9 (and mutants) and 4.8 nM nsp12 (and mutants). Reactions were started with 1 mM MgCl_2_ or MnCl_2_, 200 μM [α-^32^P] ATP, [α-^32^P] UTP, [α-^32^P] GTP, or [α-^32^P] CTP (specific radioactivity = 1000 cpm/pmol). The reactions were incubated at 37°C for 5 minutes and stopped by adding 2 μL of 500 mM EDTA, followed by addition of 5X SDS-PAGE sample buffer with 10% β-ME and incubated for 3 minutes at 95°C. Reaction products were resolved by SDS-PAGE on a 4-20% gradient gel and visualized by staining with Coomassie Brilliant Blue. The ^32^P signal was detected via autoradiography and scintillation counting.

### Nsp9 NMPylation kinetics

NMPylation reactions were carried out in a 20 μL reaction containing 50 mM Tris (pH 7.5), 5 mM KCl, 1 mM DTT, 16 μM nsp9, and 4.8 nM nsp12. Reactions were started by adding MnCl_2_ and [α-^32^P] ATP, CTP, GTP, or UTP as indicated. The final concentration in the reaction was 0.5 to 200 μM (specific radioactivity = ~5000 cpm/pmol) of the indicated nucleotide triphosphate and 1 mM MnCl_2_. The reactions were incubated at 37°C for 5 minutes and stopped by adding 2 μL of 500 mM EDTA, followed by addition of 5X SDS-PAGE sample buffer + β-ME and boiling for 2-5 minutes. Reaction products were resolved by SDS-PAGE on a 4-20% gradient gel and visualized by staining with Coomassie Brilliant Blue. Incorporation of ^32^P was quantified by excising the nsp9 bands from the gel and scintillation counting. Background radioactivity was subtracted from each measurement. Rate measurements were fit to Michaelis-Menten kinetic models and K_m_ and V_max_ for substrates were calculated by nonlinear regression using Prism 9.3.0 for macOS (GraphPad Software, San Diego, California USA, www.graphpad.com).

### NMR

For NMR studies, non-isotopically enriched AMPylated nsp9 was dissolved in 50mM Tris buffer at pH 7.5, 150mM NaCl, 1mM DTT and 10% D_2_O for spectrometer locking. The final protein concentration of this solution was 0.5mM. A total volume of 500uL was then used with a 5mm NMR tube to record all the spectra.

All NMR experiments were run on a Bruker Avance III spectrometer operating at 600MHz (1H) and equipped with a 5mm proton-optimized quadruple resonance cryogenic probe. The temperature of the sample was regulated at 308K throughout data collection.

A one-dimensional (1D) 31P spectrum was recorded with 8192 scans and a repetition delay of 1.5sec for a total collection time of 3.5 hours. The 31P spectral window and offset were set to 17ppm and 2.6ppm, respectively. Waltz16 decoupling was used on 1H during 31P acquisition.

To observe contacts between 31P and the nearest 1H nuclei, a two-dimensional (2D) 1H,31P-HSQC spectrum was recorded with 1024 and 22 complex points in the direct 1H and indirect 31P dimensions, respectively. The spectral window and offset were set to 16.7ppm and 4.7ppm for the 1H dimension and 3.4ppm and 2.6ppm for the 31P dimension, respectively. Each FID was accumulated with 1536 scans with a repetition delay of 1sec for a total recording time of approximately 21 hours.

A 2D 1H,31P-HSQC-TOCSY spectrum was recorded using similar spectral window and offset parameters for the 1H and 31P dimensions as the HSQC spectrum described above. A 60ms long 1H-1H TOCSY pulse train using a DIPSI-2 sequence and a field strength of 10KHz was tagged at the end of the HSQC sequence to observe signals from 1H nuclei that are further away from 31P. Given the lower sensitivity of this experiment, each FID was accumulated with 4096 scans and a repetition delay of 1sec was used for a total recording time of 2 days and 14 hours.

Using a similar pulse sequence, a 2D 1H,1H-HSQC-TOCSY spectrum was also recorded by evolving the indirect 1H dimension instead of 31P. The spectral window for the 1H indirect dimension was set to 4.2ppm, while the offset was maintained at 4.7ppm as for the direct 1H dimension. 40 complex points were recorded for then indirect 1H dimension, using 2048 accumulations for each FID and a repetition delay of 1sec for a total recording time of 2 days and 14 hours.

All 2D spectra were processed using NMRPipe ^37^ and analysed with NMRFAM-SPARKY ^38^.

### Intact mass analysis

Protein samples were analysed by LC/MS, using a Sciex X500B Q-TOF mass spectrometer coupled to an Agilent 1290 Infinity II HPLC. Samples were injected onto a POROS R1 reverse-phase column (2.1 x 30 mm, 20 μm particle size, 4000 Å pore size) and desalted. The mobile phase flow rate was 300 μL/min and the gradient was as follows: 0-3 min: 0% B, 3-4 min: 0-15% B, 4-16 min: 15-55% B, 16-16.1 min: 55-80% B, 16.1-18 min: 80% B. The column was then re-equilibrated at initial conditions prior to the subsequent injection. Buffer A contained 0.1% formic acid in water and buffer B contained 0.1% formic acid in acetonitrile.

The mass spectrometer was controlled by Sciex OS v.1.6.1 using the following settings: Ion source gas 1 30 psi, ion source gas 2 30 psi, curtain gas 35, CAD gas 7, temperature 300 °C, spray voltage 5500 V, declustering potential 80 V, collision energy 10 V. Data was acquired from 400-2000 Da with a 0.5 s accumulation time and 4 time bins summed. The acquired mass spectra for the proteins of interest were deconvoluted using BioPharmaView v. 3.0.1 software (Sciex) in order to obtain the molecular weights. The peak threshold was set to ≥ 5%, reconstruction processing was set to 20 iterations with a signal-to-noise threshold of ≥ 20 and a resolution of 2500.

### RNAylation assays

RNAylation reactions were typically carried out in a 10 μL volume containing 50 mM Tris (pH 7.5), 5 mM KCl, 1 mM DTT, 0.4 μg yeast inorganic pyrophosphatase, 20 μM nsp9, and 2 μM nsp12. Reactions were started by adding MnCl_2_ and 5′-pppRNA^LS10^ to a final concentration of 1 mM and 100 μM, respectively. Reactions were incubated at 37°C for the indicated time points and stopped by addition of 5X SDS-PAGE sample buffer + β-ME and boiling the samples for 5 minutes. Reaction products were resolved by SDS-PAGE on a 4-20% gradient gel and visualized by Coomassie staining.

For the time course comparing the RNAylation of nsp9 N1A and N2A mutants (i.e. Fig. 2d), reactions were performed as above, except with 2.4 μM nsp12. At each indicated time point, reactions were stopped by addition of 5X SDS-PAGE sample buffer + β-ME and boiling the samples for 5 minutes. Reaction products were resolved by SDS-PAGE on a 4-20% gradient gel and visualized with Coomassie staining.

For reactions testing RNA length specificity (i.e. Fig. 2e), 7.5 μL of a reaction master mix containing nsp9, nsp12, and yeast inorganic pyrophosphatase was added to 2.5 μL of start mix consisting of MnCl_2_ and the indicated 5′-pppRNA. The final reaction conditions were as follows: 50 mM Tris pH 7.5, 5 mM KCl, 1 mM DTT, 0.4 μg yeast inorganic pyrophosphatase, 20 μM nsp9, 2 μM nsp12, 1 mM MnCl_2_, and 100 μM of the indicated RNA. Reactions were incubated for 30 minutes at 37°C, then stopped by addition of 5X SDS-PAGE sample buffer + β-ME and boiling the samples for 5 minutes. Reaction products were resolved by SDS-PAGE on a 4-20% gradient gel and visualized by Coomassie staining.

For RNAylation reactions comparing different RNA sequences (i.e. Fig. 2f) or nsp9 mutants (i.e. Fig. 5h), reactions were performed as above, except using 1 μM nsp12. Reactions were incubated for 5 minutes and stopped by addition of 5X SDS-PAGE sample buffer + β-ME and boiling the samples for 5 minutes. Reaction products were resolved by SDS-PAGE on a 4-20% gradient gel and visualized by Coomassie staining.

### Purification of nsp9-pRNA^LS10^ species

Purified native nsp9 (0.8 mg/mL, 65 μM) was incubated at room temperature overnight with 130 μM of 5′-pppRNA^LS10^ and ~0.8 μM of nsp12 in presence of 0.05 mg/ml yeast inorganic pyrophosphatase and 1 mM MnCl_2_, in the reaction buffer (50 mM Tris 7.5, 5 mM KCl, 1 mM DTT). The samples were clarified by centrifugation to remove any precipitate and applied directly onto a Capto HiRes Q 5/50 column (Cytiva) equilibrated in 50 mM Tris 8.0, 50 mM NaCl, 1 mM DTT. An elution gradient of 0-50% with 1 M NaCl was applied over 30 column volumes. Under these conditions, RNA and nsp9-pRNA^LS10^ bound the column and unmodified nsp9 did not. nsp9-pRNA^LS10^ and unreacted RNA^LS10^ eluted as a peak doublet around 70 mS/cm. Fractions were pooled, and further purified over Superdex 75 increase 10/300 GL (50 mM Tris 8.0, 300 mM NaCl, 1 mM DTT), separating nsp9-pRNA^LS10^ from unreacted RNA^LS10^, and nsp12. nsp9-pRNA^LS10^ was quantified by spectrophotometry with an estimated extinction coefficient of ϵ_260_=130,650 M^−1^cm^−1^. We also generated nsp9-pRNA^LS10^ in the absence of inorganic pyrophosphatase. This nsp9-pRNA^LS10^ was used in control reactions to test for PP_i_ hydrolysis, necessary to ensure that PPi mediated deRNAylation reactions shown in Fig. 3e do not suffer from pyrophosphate hydrolysis. The results were like those presented in the Figure, thus confirming that there was no contaminating inorganic pyrophosphatase in the assays.

### DeRNAylation of nsp9-pRNA^LS10^

DeRNAylation reactions were typically performed in a 10 μL reaction volume consisting of 50 mM Tris pH 7.5, 5 mM KCl, 1 mM DTT, 20 μM nsp9-pRNA^LS10^, 1 μM nsp12, 1 mM MgCl_2_, and 500 μM GDP. Reactions were started by adding MgCl_2_/GDP and incubated at 37°C for 5-60 minutes as indicated. Reactions were stopped by addition of 5X SDS-PAGE sample buffer + β-ME and boiling the samples for 5 minutes. Reaction products were resolved by SDS-PAGE on a 4-20% gradient gel and visualized with Coomassie staining.

For deRNAylation reactions comparing various nucleotide triphosphates (NTP) and nucleotide diphosphates (NDP), reactions were performed in 10 μL volume consisting of 50 mM Tris pH 7.5, 5 mM KCl, 1 mM DTT, 20 μM nsp9-pRNA^LS10^, 500 nM nsp12, 1 mM MgCl_2_, and 500 μM of the indicated NTP or NDP. Reactions were incubated for 5 minutes at 37°C and stopped by addition of 5X SDS-PAGE sample buffer with β-ME and boiling for 5 minutes. Reaction products were resolved by SDS-PAGE on a 4-20% gradient gel and visualized with Coomassie staining.

### Generation of [α-^32^P]-GDP using nsp13

To generate [α-^32^P]-GDP from [α-^32^P]-GTP, 0.3-1 mM of [α-^32^P]-GTP (specific activity ~2000 cpm/pmol) was incubated with 0.5-1 mg/mL nsp13 (and in some cases yeast cet1 NTPase) in 20 μL reaction buffer (depending on amount needed) consisting of 50 mM Tris (pH 7.5), 5 mM KCl, 1 mM DTT, 2 mM MgCl_2_. Reactions were started by addition of enzyme and allowed to proceed for 30 minutes at 37°C. Following the 30-minute incubation, reactions were boiled at 95°C for 5 minutes to inactivate nsp13 or cet1.

### Generation of radiolabelled GpppA-RNA^LS10^ from nsp9-pRNA^LS10^ and [α-^32^P]-GDP

Reactions were performed in a 10 μL volume containing 50 mM Tris pH 7.5, 5 mM KCl, 1 mM DTT, 15 μM nsp9-pRNA^LS10^, 377 nM nsp12, 1 mM MgCl_2_, and 500 μM [α-^32^P]-GDP (specific radioactivity = ~2000 cpm/pmol). Reactions were started by addition of [α-^32^P]-GDP/MgCl_2_ mixture (generated as described above) and incubated for 30 minutes at 37°C. As a control, VCE was used but with [α-^32^P]-GTP. VCE assays were generally performed as described in the NEB Capping Protocol (M2080) with the following modifications: the reaction contained 20 μM of 5’-pppRNA^LS10^, 500 μM [α-^32^P]-GTP (specific activity ~2000cpm/pmol), and did not contain SAM. Reactions were stopped by the addition of 2X TBE-Urea sample buffer, boiled for 5 minutes, and resolved by UREA-PAGE (20%).

### GDP inhibition of RNAylation (one pot capping assays)

Reactions were performed in 50 mM Tris pH 7.5, 5 mM KCl, 1 mM DTT, 1 mM MgCl_2_, 1 mM MnCl_2_, and contained 20 μM nsp9, 2 μM nsp12, and 100 μM 5’-pppRNA^LS10^ in 20 μL volume. The [α-^32^P]-GDP was prepared as described earlier (using 400 μM GTP, [α-^32^P]-GTP at specific activity ~1,500 cpm/pmol) and diluted to final reaction concentrations in the range of 6.25-100 μM. Reactions were started by the addition of nsp12. GDP was added either before the addition of nsp12 (t=0), or after 30 minutes of preincubation. After an additional 30 minutes, the reactions were split in half and stopped by the addition of 5x SDS-PAGE or 2x Formamide loading dyes and the products were analysed by 4-20% gradient SDS-PAGE gel, or 15% 19:1 TBE UREA-PAGE gel, respectively.

### LC-MS/MS analysis of GpppA

De-RNAylation reactions (in triplicate) were performed in 20 μL of buffer solution containing 50 mM Tris pH 7.5, 5 mM KCl, 20 μM nsp9-pRNA^LS10^, 2 μM wild-type nsp12 or the D218A mutant, 1 mM MgCl_2_, and 100 μM GDP. Reactions were started by adding MgCl_2_ and GDP and allowed to proceed for 1 hour at 37°C. After the 1-hour incubation, the reactions were supplemented with 2 μL of 10X P1 buffer and 1 μL of Nuclease P1 enzyme and allowed to proceed for an additional 30 minutes at 37°C. Reactions were stopped by boiling for 5 minutes and submitted for LC-MS/MS analysis.

For standards, 20 μL of blank reaction buffer (50 mM Tris pH 7.5, 5 mM KCl, 1 mM DTT) plus 40 μL blank reaction buffer containing 0.33 μM (final) m7GpppA (m7G(5′)ppp(5′)A RNA Cap Structure Analog, New England Biolabs, S1405S) as an internal standard (IS) was spiked with varying concentrations of GpppA (New England Biolabs, S1406L). Reaction samples (20 μL) were diluted with blank reaction buffer, containing 0.33 μM (final) m7GpppA IS, at 1:2 to a total volume of 60 μL. Standards and samples were mixed with 60 μL of 100% methanol, vortexed, and then spun for 5 min at 16,100 x g. Supernatant was removed and analysed by LC-MS/MS using a Sciex (Framingham, MA) QTRAP® 6500+ mass spectrometer coupled to a Shimadzu (Columbia, MD) Nexera X2 LC. GpppA was detected with the mass spectrometer in positive MRM (multiple reaction monitoring) mode by following the precursor to fragment ion transition 772.9 → 604.0. A Thermo Scientific BioBasic AX column (2.1 x 50 mm, 5 micron packing) was used for chromatography with the following conditions: Buffer A: 8:2 dH2O:Acetonitrile + 10 mM ammonium acetate, pH 6, Buffer B: 7:3 dH_2_O:Acetonitrile + 1 mM ammonium acetate, pH 10.5, 0.5 mL/min flow rate, 0-1 min 0%B, 1-2.5 min gradient to 35%B, 2.5-5 min 35%B, 5-7 min gradient to 65%B, 7-10 min 65%B, 10-10.5 min gradient to 100%B, 10.5-15 min 100%B, 15-15.5 min gradient to 0%B, 15.5-20.5 min 0%B. m7GpppA (transition 787.1 → 508.0) was used as an internal standard. Peak areas were determined and data were further analysed using the Sciex Analyst 1.7.2 software package. Back-calculation of standard curve samples were accurate to within 15% for 100% of these samples at concentrations ranging from 0.001 μM to 10 μM. A limit of detection (LOD) was defined as a level three times that observed in blank reaction buffer and the limit of quantitation (LOQ) as the lowest point on the standard curve that gave an analyte signal above the LOD and within 20% of nominal upon back-calculation. The LOQ for GpppA was 0.005 μM.

### Methyltransferase assays

In 10 μL reactions, 40 μM nsp9-pRNA^LS10^ was incubated with 2 μM nsp12 in presence of 1 mM MgCl_2_ and 100 μM [^32^P]GDP (generated as above) for 60 min at 37 °C. Reactions were filled to 15 μL with nsp14 and SAM (final 0.05 mg/mL and 100 uM respectively), and incubated for another 30 minutes. Treatment with nsp10/16 can be done concurrently with nsp14, however nsp10/14 complex partially processes RNA^LS10^, resulting in a mobility shift ^39^. Thus, prior to addition of nsp10 and nsp16, nsp14 exonuclease activity was removed by heat inactivation (5 minutes at 95 °C). For 2’-O methylation, reactions were supplemented with nsp10/16 and fresh SAM to final concentrations of 0.05 mg/mL nsp10, 0.05 mg/mL nsp16, 100 μM SAM in final 20 μL volume. Vaccinia reactions were conducted as per manufacturer’s instructions. Reactions were stopped by adding 2x formamide loading dye and were separated on 20% TBE-UREA polyacrylamide gels (19:1). Radioactivity was visualized by autoradiography and RNA by toluidine staining. For TLC analysis, bands with detectible ^32^P signal were excised, fragmented, and incubated overnight at 55°C in elution buffer (1 M Ammonium acetate, 0.2% SDS, 20 mM EDTA), rotating top-over-bottom. Solutions were filtered using 0.22 μm centrifugal filters, supplemented with 23 ug Glyco Blue co-precipitant (Invitrogen) and precipitated for 1 hr at −20°C by addition of isopropyl alcohol to a final concentration of 60%. The pellets were washed once with 70% EtOH and reconstituted in 10 μL of P1 buffer with P1 nuclease (NEB). After 30 minutes at 37°C, reactions were supplemented with Quick CIP (NEB) and rCut Smart buffer, to a final volume of 12 uL. After 30 min of further incubation, reactions were spotted onto PEI-Cellulose F TLC plates and resolved in 0.4 M Ammonium Sulfate mobile phase. Beforehand, TLC plates were prepared by development in water, removing yellow discoloration. The ^32^P signal was detected by autoradiography, and compared with cold standards of GTP, GDP, GpppA and ^m7^GpppA detected by absorption of plate fluorescence, excitable with a UV lamp λ=265 nm. The position of ^m7^GpppA_2’-OMe_ was determined from the Vaccinia capping enzyme and Vaccinia 2’-O-Methyltransferase control reaction.

Reactions with ^14^C-labelled SAM were conducted as above, with two differences: cold GDP was used at 100 μM (Millipore Sigma, G7127), and 55 μM [^14^C ]SAM (Perkin Elmer), SAM was used at the supplied radioactivity of 52.6 mCi/mmol (~117 cpm/pmol), with no further dilution using cold SAM.

### GST-eIF4E pulldown of ^7Me^GpppA-RNA

Capping reactions were set up in 20 μL and contained 2 μM nsp12, 0.04 mg/ml nsp14 WT or D331A, 30 μM nsp9-pRNA^LS10^, 100 μM [α^32^P]GDP (specific radioactivity = 1,000 cpm,/pmol), 100 μM SAM, 2 mM MgCl_2_. The reaction buffer was 50 mM Tris 8.0, 5 mM KCl, 1 mM DTT. Vaccinia capping enzyme controls were performed according to manufacturer’s instructions, with the same nucleotide and SAM concentrations as nsp12 reactions. After 90 minutes of incubation at 37 °C, 15.6 ug of GST-eIF4E K119A was added, along with 15 μL of Glutathione resin (Pierce, Thermo Scientific). Reactions were filled to 700 μL with 50 mM Tris 8.0, 150 mM NaCl, 1 mM DTT, and nutated for 1 hour. The resin was washed 3x with 500 μL of 50 mM Tris 8.0, 150 mM NaCl, 1 mM DTT, and radioactive signal was quantified by scintillation counting.

### Cryo-EM grid preparation

To form nsp12/7/8 core complex (RTC), native nsp12, nsp7 and nsp8 were incubated in 1:2:4 molar ratio and run over Superdex 200 increase 10/300 GL to separate unassociated monomers. The purified complex was concentrated using spin concentrators (Amicon 10k MWCO, Sigma-Millipore), and quantified by spectrophotometry. A 3x molar excess of nsp9 over RTC was added, followed by 0.05 mM final DDM detergent immediately prior to freezing. Final concentration of nsp12/7/8 was 2 mg/mL. Buffer contained 50 mM Tris 7.5, 150 mM NaCl, 1 mM DTT, 2 mM MnCl_2_, 1 mM UMP-NPP. Copper Quantifoil 1.2/1.3 mesh 300 grids were used to freeze 3.5 μL of sample at 100% relative humidity using Vitrobot mk. IV (Thermofisher).

### Cryo-EM data collection

Prior to data collection, sample grids were screened on a Talos Artica microscope at the Cryo Electron Microscopy Facility (CEMF) at UT Southwestern. Cryo-EM data of NSP12/7/8/9 complex were collected on a Titan Krios microscope at Cryo-Electron Microscopy Facility (CEMF) at UT Southwestern Medical Center, with the post-column energy filter (Gatan) and a K3 direct detection camera (Gatan), using SerialEM ^40^. 4,770 movies were acquired at a pixel size of 0.55 A in super-resolution counting mode, with an accumulated total dose of 54 e-/Å^2^ over 50 frames. The defocus range of the images was set to be −1.0 to −2.5 μm.

### Image processing and 3D reconstruction

Unless described otherwise, all datasets were processed with Relion ^41^. Movies were aligned and summed using MotionCor2 ^42^, with a downsampled pixel size of 1.09 Å. The CTF parameters were calculated using Gctf ^43^, and images with estimated CTF max resolution better than 5 A ° were selected for further processing. 4,196,086 particles were picked using crYOLO ^44^ from 4,757 images, and extracted with a re-scaled pixel size of 2.19 Å. 663,999 particles were selected and re-extracted after multiple rounds of 2D and 3D classifications in Relion with the original pixel size of 1.09 Å. An additional round of 3D classification was carried out, followed by particle reduction with a homemade script to remove particles from dominant orientations. The remaining 89,945 particles were subjected to 3D refinement, CTF refinement and particle polishing sequentially. A final round of 3D classification with a reference mask led to 39,985 particles, which were then imported into cryoSPARC ^45^ for one round of non-uniform refinement. The map resolution was reported at 3.18 Å from cryoSPARC with the gold standard FSC method.

### Nsp5 cleavage reactions

Concentrated protein samples were diluted in cleavage buffer (50 mM Tris, pH 7.4; 150 mM NaCl; 5% glycerol) and each reaction was performed in a total volume of 10 μL. Initial experiments measured the nsp5 concentration dependence of the nsp8-nsp9 cleavage reaction. To measure time dependence, nsp5 (2.5 μM final concentration) was added to the nsp8-nsp9 fusion protein (12.5 μM final concentration). Reactions were incubated at 37 °C for varying amounts of time (0 to 80 minutes) and terminated by boiling the samples for five minutes in the presence of SDS-PAGE loading buffer. Reaction products were resolved on a 4-20% gradient tris-glycine gel and products were visualized by Coomassie staining.

### Model building and refinement

Model was build using PDB 7CYQ as a template ^21^. Model was manually rebuilt into the map using Coot ^46^, and refined using Phenix real space refinement ^47^. Model validation was performed using MolProbity software ^48^.

### Bioinformatics

A representative subset of NiRAN domain sequences, provided as a multiple alignment in the original NiRAN publication ^5^, was supplemented by additional sequences used in this study (SARS-CoV-2, OC43, 229E strains). The human SELO sequence was added according to a FATCAT structural alignment ^49^ between SARS-CoV-2 nsp12 and bacterial SelO (PDB identifiers 7cyq and 6eac). The alignment was visualized using the ESPript server ^50^.

### SARS-CoV-2 infection experiments

#### Plasmid Construction

To generate recombinant SARS-CoV-2 expressing ZsGreen mutants, the infectious clone pCC1-4K-SARS-CoV-2-Wuhan-Hu-1-ZsGreen was used as the parental backbone ^8^. To generate nsp9 (N1A, N1D, N2A) and nsp12 (K73A) mutants, mutations were introduced by overlap extension PCR. Briefly, 2 fragments for each mutant were created by PCR using Ex Taq DNA Polymerase (Takara). These fragments shared homology at the 3’ end of Fragment 1 and 5’ end of Fragment 2. The resulting 2 fragments were then used as a template for a third fragment to PCR a full-length amplicon containing the mutation flanked by Pac1 and MluI sites on the ends. Mutations were confirmed by DNA sequencing. To generate nsp12 mutants (D218A, D760A), a SARS-CoV-2 shuttle vector (ps1180.SARS-CoV-2-shuttle) was created using ps1180.delXhoISacII plasmid as the backbone. Using Gibson cloning, a restriction enzyme linker that contained unique restriction sites specific to the SARS-CoV-2 genome was inserted. Smaller fragments of the SARS-CoV-2 genome were digested from the pCC1-4K-SARS-CoV-2-Wuhan-Hu-1-ZsGreen plasmid and ligated into ps1180.SARS-Cov-2-shuttle plasmid to create 3 new plasmids (MluI/SacI fragment for D218A region; SacI/Bsu36I fragment for D760A region). gBlocks containing mutations were synthesized by IDT and introduced into the SARS-CoV-2 shuttle vector by Gibson Assembly following standard protocols. To reassemble the full length parental pCC1-4K-SARS-CoV-2-Wuhan-Hu-1-ZsGreen containing new mutants, pCC1-4K-SARS-CoV-2-Wuhan-Hu-1-ZsGreen was digested with PacI/MluI, MluI/SacI, and SacI/Bsu36I restriction enzymes. A roughly 28-35 kb fragment was purified for each digest. PCR Amplicons (nsp9^N1A^, nsp9^N1D^, nsp9^N2A^, nsp12^K73A^) were digested with PacI/MluI and 5.3 kb fragment was purified. The SARS-CoV-2 shuttle plasmids were digested as follows: nsp12^D218A^ (MluI/SacI releasing 1.4 kb fragment); nsp12^D760A^ (SacI/Bsu36I/PvuI releasing 3 kb fragments). All fragments were purified using QIAexII Gel Purification Kit following standard protocol (Qiagen). Fragments were ligated together at a 3:1 ratio overnight. Ligated DNA was precipitated using 7.5 M Ammonium acetate, Glycogen, and Isopropanol, followed by an ethanol wash. The DNA was electroporated into TransforMAX EPI300 Electro competent *E. coli* (Lucigen). An overlapping 8-fragment PCR strategy was used to verify individual colonies by colony PCR. Confirmed colonies were grown in 10 ml Tryptic Soy Broth (TSB; Sigma) containing 12.5 μg/ml Chloramphenicol for 6-8 hours, shaking at 37°C. A 10 ml culture was inoculated into 100 ml TSB/Chloramphenicol culture and incubated overnight, shaking at 37°C. Overnight culture was diluted 1:5 into fresh TSB/Chloramphenicol containing 0.1% Arabinose and incubated an additional 5 hrs. Bacteria was pelleted and DNA was isolated using a homemade midi prep protocol followed by Machery-Nagel NucleoBond Xtra Midi Kit (Fisher). Full-length infectious clone plasmid was confirmed by restriction digestion and 8-fragment PCR. Oligonucleotides used are shown in Table S3.

#### Virus production

To generate virus from DNA-based infectious clones, 3 μg of plasmid were transfected into 2 individual 6 wells of 400,000 BHK-21J cells using X-treme Gene9 Transfection Reagent (Sigma). Three days post transfection, the supernatant from 2 individual wells was combined and 3 ml was transferred to a T25 flask containing 1 x 10^6^ VeroE6-C1008-TMPRSS2 cells and 2 ml serum-free MEM. After 4 days, 250 μl supernatant was added to 750 μl TriReagent for RNA extraction and RT-qPCR. T25 flasks were fixed with 4% paraformaldehyde, imaged on Nikon Eclipse Ti and processed with ImageJ.

#### RNA Extraction/RT-qPCR

RNA was isolated using the Direct-zol RNA mini prep kit following manufacturer’s instructions (ZymoResearch). A 20 μl reaction contained 5 μl RNA, 5 μl TaqMan Fast Virus 1-Step Master Mix, and 1.8 μl SARS-CoV-2 primer/probe set containing 6.7 μM each primer/1.7 μM probe (final concentration of primer/probe were 600 nM/150 nM probe). SARS-CoV-2 primers and probe were designed as recommended by the Center for Disease Control (https://www.cdc.gov/coronavirus/2019-ncov/lab/rt-pcr-panel-primer-probes.html). All oligonucleotides were synthesized by LGC Biosearch Technologies. RT was performed at 50°C for 5 minutes, followed by inactivation at 95°C for 2 minutes, and 40 cycles of PCR (95°C for 3 seconds, 60°C for 30 seconds) on a QuantStudio 3 (Applied Biosystems).

#### Cells

BHK-21J cells (a generous gift from C. Rice) were grown in MEM (Gibco) supplemented with 10% FBS and 1X NEAA. VeroE6-C1008 cells (ATCC) were transduced with lentiviral vector SCRBBL-TMPRSS2, selected and maintained in MEM supplemented with 10% FBS, 1X NEAA, and 8 μg/ml Blasticidin.

## Extended Data

**Extended Data Fig. 2.**
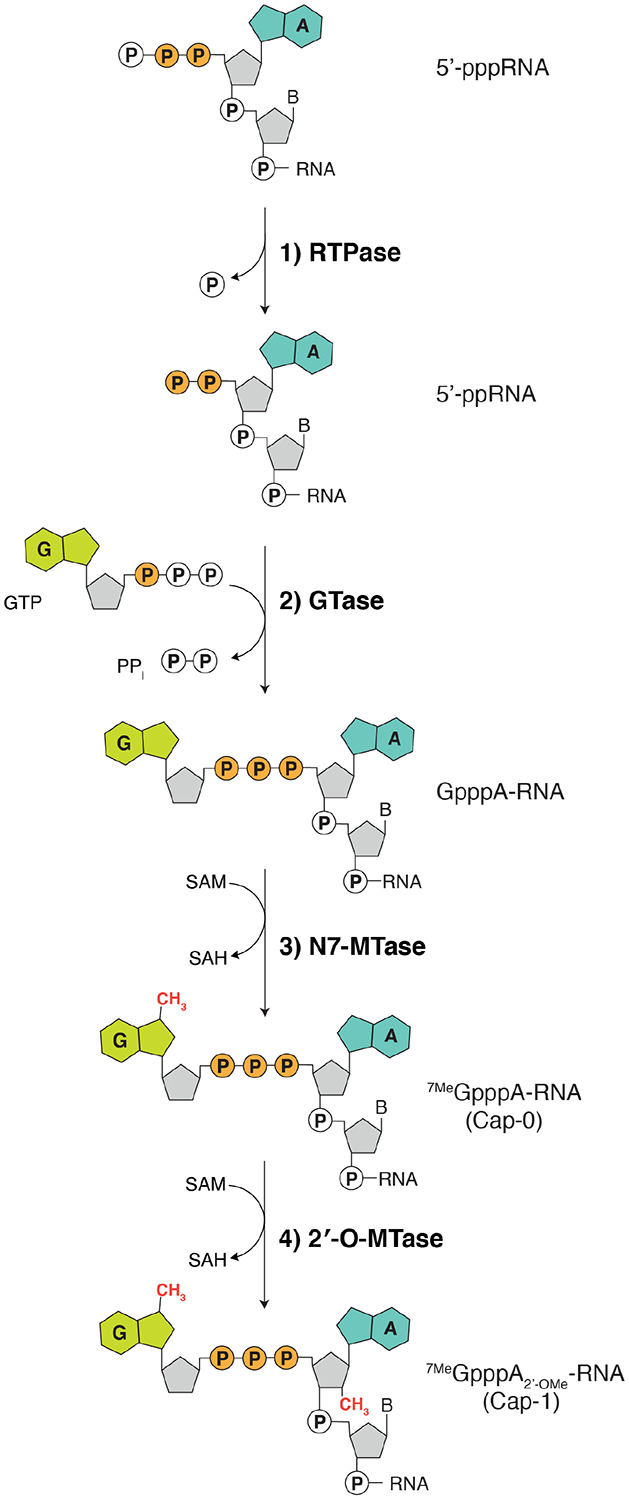
The canonical eukaryotic mRNA capping mechanism. The ^7Me^GpppA_2’OMe_ cap on eukaryotic RNA is formed co-transcriptionally by four enzymes: **1)** an RNA triphosphatase (RTPase), which removes the γ-phosphate from the nascent 5’-triphosphorylated RNA (5’-pppRNA) to yield a 5’-diphosphorylated RNA (5’-ppRNA); **2)** a guanylyltransferase (GTase), which transfers the GMP moiety from GTP to the 5’-ppRNA to form the core cap structure GpppN-RNA; **3)** a (guanine-N7)-methyltransferase (N7-MTase), which methylates the cap guanine at the N7 position; and **4)** a (nucleoside-2′-*O*)-methyltransferase (2′-O-MTase), which methylates the ribose-2’-OH position on the first nucleotide of the RNA. B denotes any base; GTP, Guanosine triphosphate; GDP, Guanosine diphosphate; PP_i_, pyrophosphate; SAM, S-Adenosyl methionine.

**Extended Data Fig. 3.**
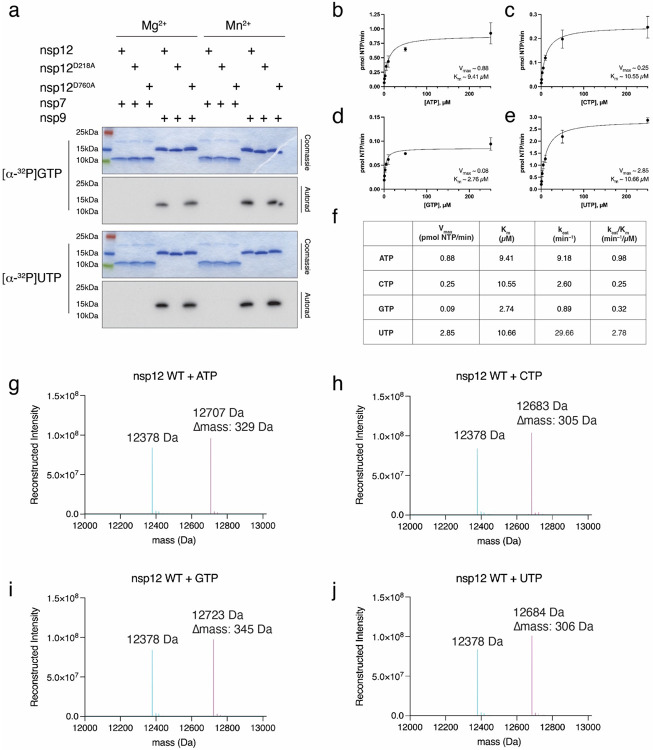
The NiRAN domain NMPylates nsp9. **a.** Incorporation of α-^32^P from [α-^32^P]GTP or [α-^32^P]UTP into nsp7 or nsp9 by WT nsp12, the NiRAN mutant (K73A, D218A), or the polymerase mutant (D760A). Reactions were performed in the presence of Mg^2+^ or Mn^2+^ and the products were resolved by SDS-PAGE and visualized by Coomassie staining (top) and autoradiography (bottom). **b-e**. Kinetic analysis depicting the concentration dependence of (**b**) ATP, (**c**) CTP, (**d**) GTP, or (**e**) UTP on the rate of nsp9 NMPylation by the NiRAN domain. K_m_ and V_max_ are indicated on the insets. Plots shown are the mean and SD of triplicate reactions. **f.** Summary of K_m_, V_max_, k_cat_, and k_cat_/K_m_ values for each NTP. **g-j**. Intact mass LC/MS spectra of unmodified nsp9 (*cyan)* overlayed with NMPylated nsp9 (*pink*) following incubation with WT nsp12 and (**g**) ATP, (**h**) CTP, (**i**) GTP, or (**j**) UTP. The observed masses are shown in the insets. The theoretical mass of unmodified nsp9 is 12378.2 Da and the theoretical increase in mass with the addition of each NMP is as follows: AMP, 329 Da; CMP, 305 Da; GMP, 345 Da; UMP, 306 Da.

**Extended Data Fig. 4.**
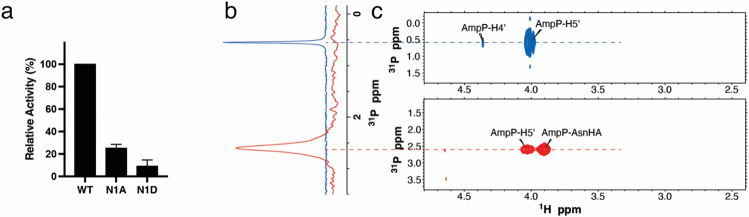
The NiRAN domain NMPylates nsp9 on the N-terminus. **a.** Quantification of reaction products from Fig. 1c depicting the relative NiRAN-dependent UMPylation activity towards nsp9 or the indicated mutants. Radioactive gel bands were excised and quantified by scintillation counting. **b.** 1D 31P spectrum of AMP-nsp9 (*red*) and AMP (*blue*) recorded in the same buffer as reference. **c.** 2D 1H, 31P-HSQC spectra of AMP (*top, blue*) and AMP-nsp9 (*bottom, red*).

**Extended Data Fig. 5.**
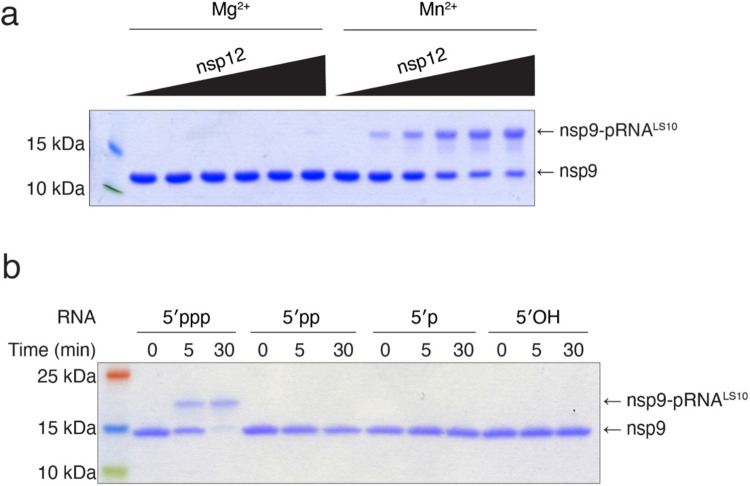
Characterization of NiRAN RNAylation activity. **a.** Incorporation of RNA into nsp9 by nsp12 (0-4 μM) in the presence of Mg^2+^ or Mn^2+^. Reaction products were analysed as in Fig. 2b. **b.** Incorporation of RNA with the indicated 5’ ends into nsp9 by nsp12. Reaction products were analysed as in Fig. 2b.

**Extended Data Fig. 6.**
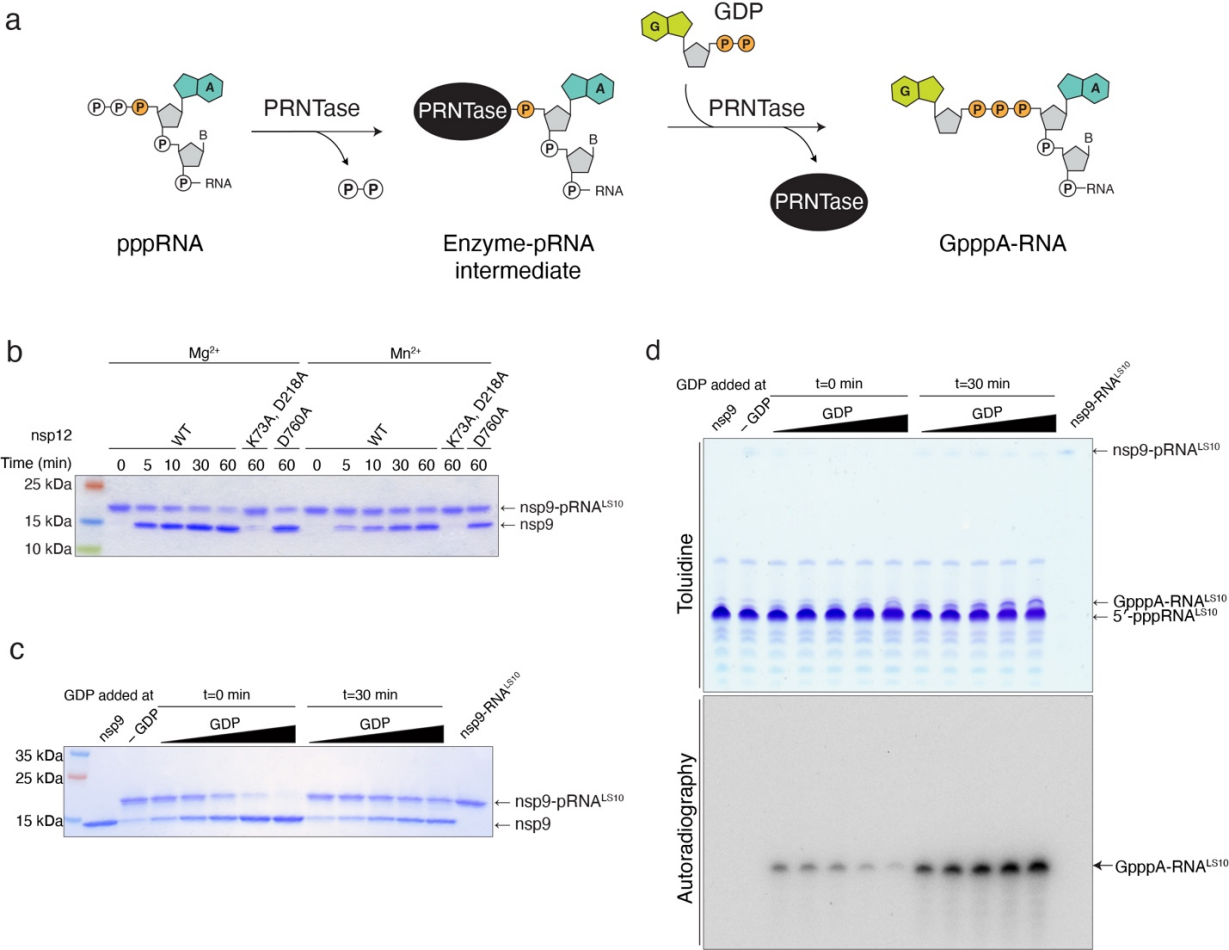
Characterization of nsp12 NiRAN GDP-PRNTase activity. **a.** Schematic representation depicting the mechanism of GpppA-RNA formation by vesicular stomatitis virus (VSV) polyribonucleotidyltransferase (PRNTase) enzyme. **b.** Time-dependent deRNAylation of nsp9-pRNA^LS10^ by WT nsp12, the NiRAN mutant (K73A, D218A), or the polymerase mutant (D760A) in the presence of GDP and either Mg^2+^ or Mn^2+^. Reaction products were analysed as in Fig. 2b. **c, d.** NiRAN-catalysed capping reactions depicting the inhibitory effect of GDP on RNAylation. Nsp9 was incubated with excess 5’-pppRNA^LS10^ in presence of nsp12 with no GDP (-GDP), or increasing concentrations (6.25-100 μM) of [^32^P]GDP added either at time zero (t =0 min), or after the RNAylation reaction was allowed to proceed for 30 minutes (t=30 min). Reaction products were analysed by SDS-PAGE **(c)**, and Urea-PAGE **(d)**.

**Extended Data Fig 7.**
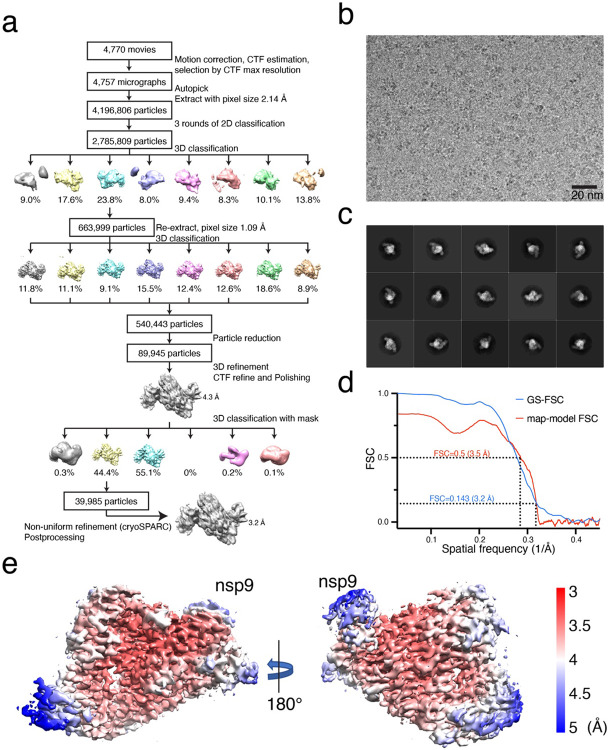
Cryo-EM analysis of the nsp7/8/9/12 complex. **a.** Flow chart representing data processing for the nsp7/8/9/12 complex. **b.** A representative micrograph of the nsp7/8/9/12 complex grids. **c.** Representative 2D classes generated by RELION 2D-classification. **d.** Gold-standard FSC curve (blue), and map-model FSC curve (red). Curves were generated by cryoSPARC and Phenix suite, respectively. **e.** Local resolution of the nsp7/8/9/12 complex calculated by RELION from final cryoSPARC half-maps. Position of nsp9 is indicated.

**Extended Data Fig 8.**
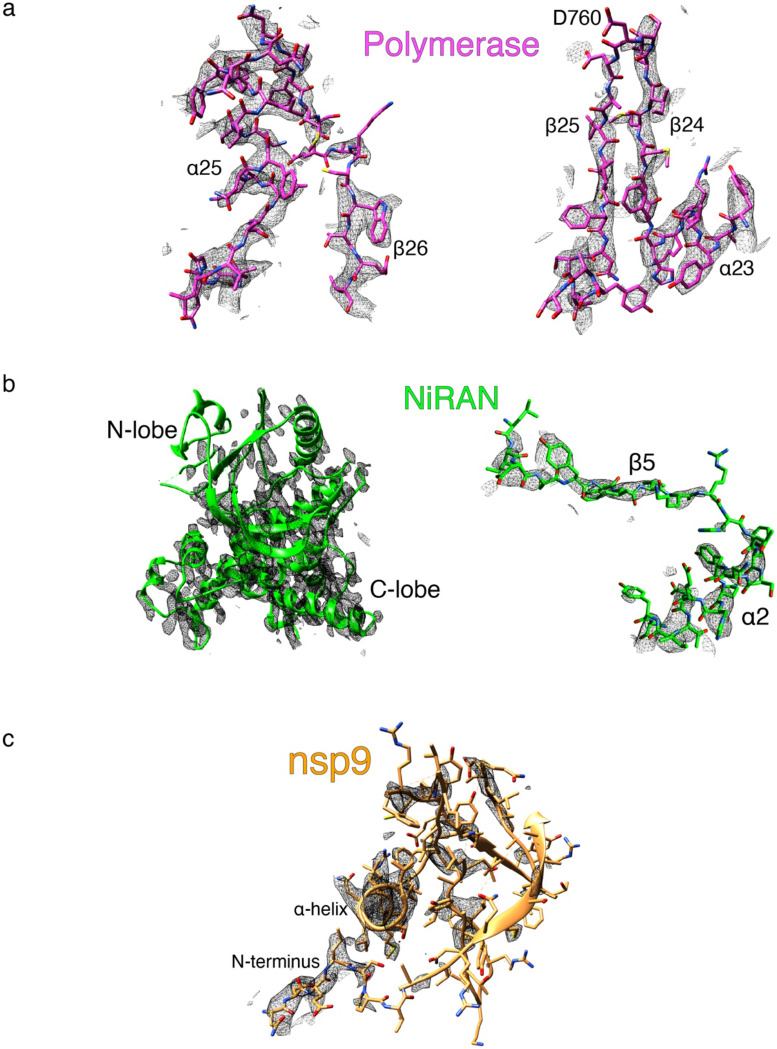
Exemplary cryo-EM density (black mesh) on (**a**) Polymerase (Magenta), (**b**) NiRAN (green) and (**c**) nsp9 (gold). Note weaker density in the N-lobe of the kinase-like NiRAN domain (left panel of **b**, compare top to bottom of the image), and poor density in nsp9 (**c**), in areas not in direct contact with the NiRAN domain.

**Extended Data Fig. 9.**
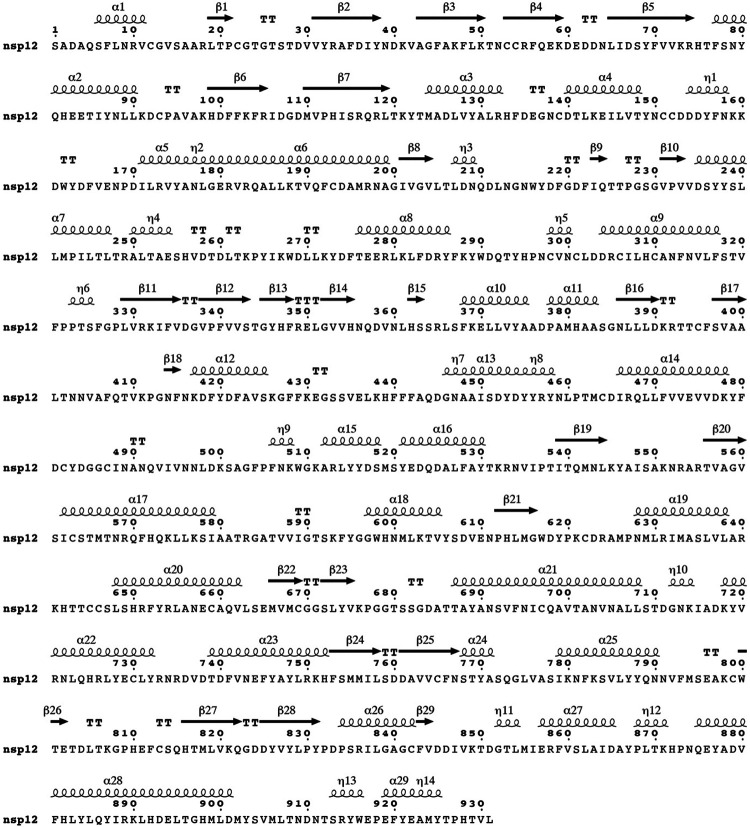
Secondary structure of nsp12. The secondary structural elements in nsp12 (from PDB ID: 7CYQ) are shown.

**Extended Data Fig. 10.**
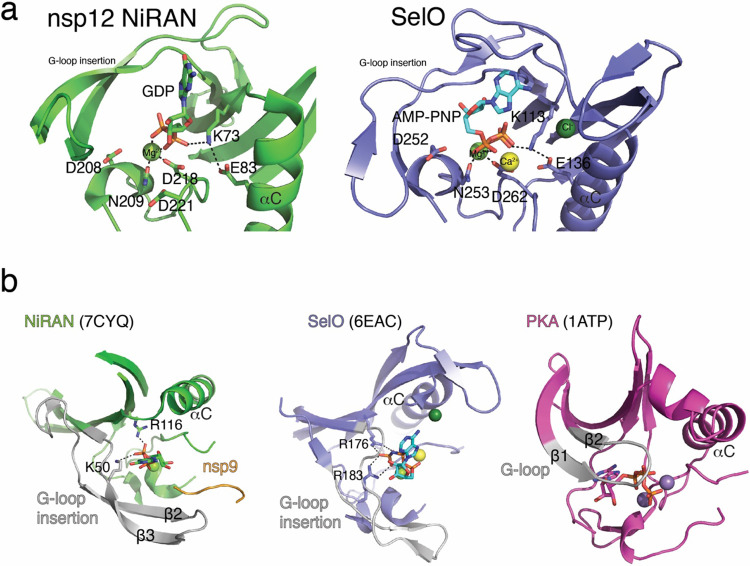
Comparison of the kinase-like domains of nsp12 and SelO. **a.** Cartoon representation comparing the NiRAN active site catalytic residues (green) to the active site residues in SelO (purple). The divalent cations are shown as spheres. **b.** Comparison of the Gly-rich loop regions in NiRAN (PDB ID: 7CYQ, left), SelO (PDB ID: 6EAC), and the canonical kinase PKA (PDB ID: 1ATP, right). Green sphere – Mg^2+^, dark green sphere – Chloride, yellow sphere – calcium, violet sphere – Mn^2+^.

**Extended Data Fig. 11.**
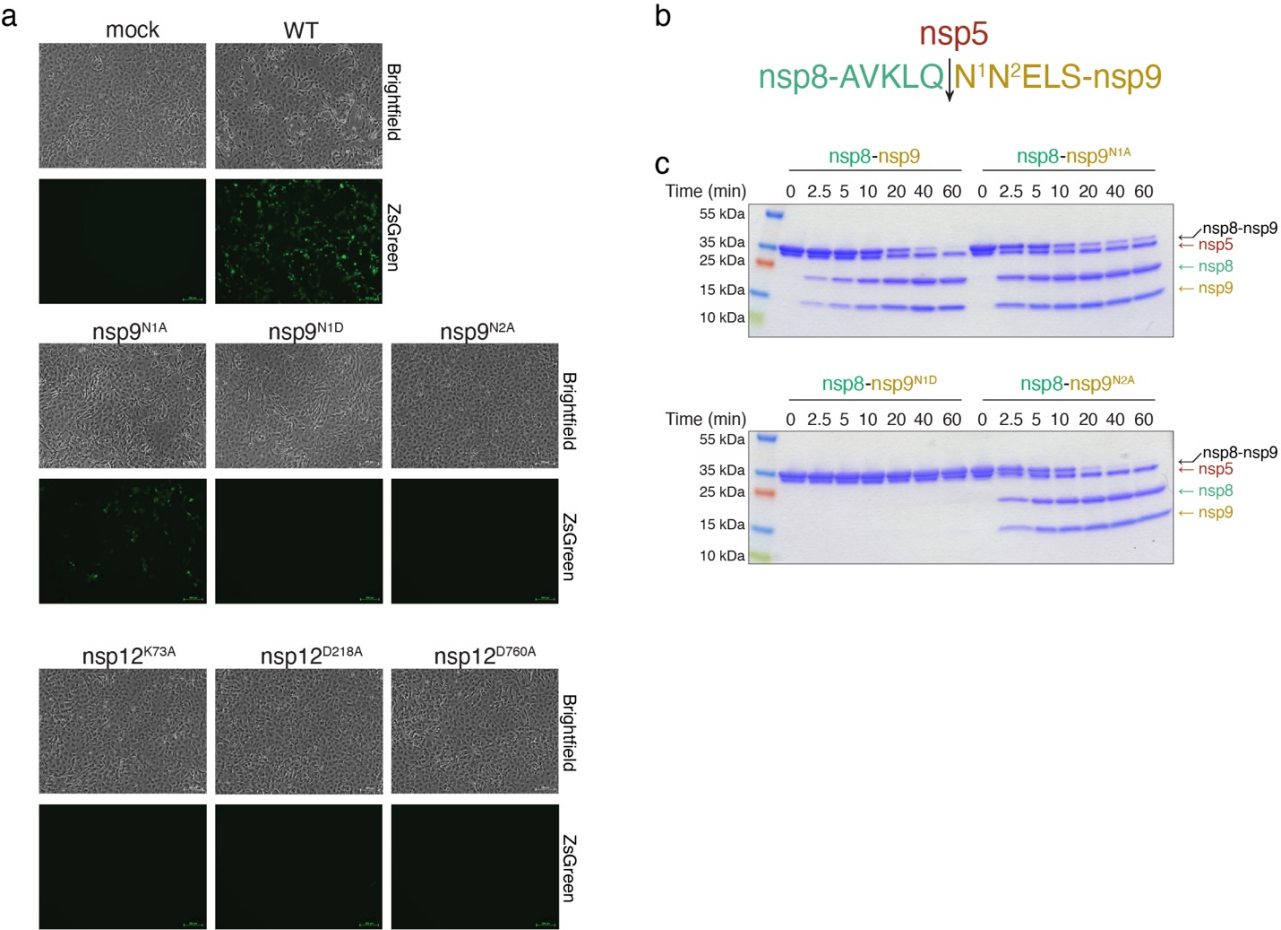
Genetic insights into RNA capping by the NiRAN domain. **a.** Microscopy images showing brightfield (upper) or fluorescence-based images (ZsGreen; lower) of SARS-CoV-2-ZsGreen production in VeroE6-C1008-TMPRSS2 cells. Mock-transfected panels were incubated with transfection reagents lacking DNA. The mutations engineered into either nsp9 or nsp12 are indicated above each set of images. Data represent one set of images from two independent biological replicates. Scale bars, 100 μm. **b.** The amino acid sequence between nsp8 (green) and nsp9 (gold) depicting the cleavage site for the nsp5 (dark red) protease. N1 and N2 of nsp9 are highlighted. The arrow denotes the location of cleavage. **c.** Time-dependent proteolysis of the nsp8-nsp9 fusion protein by nsp5. Reaction products were separated by SDS PAGE and visualized by Coomassie staining.

**Extended Data Fig. 12.**
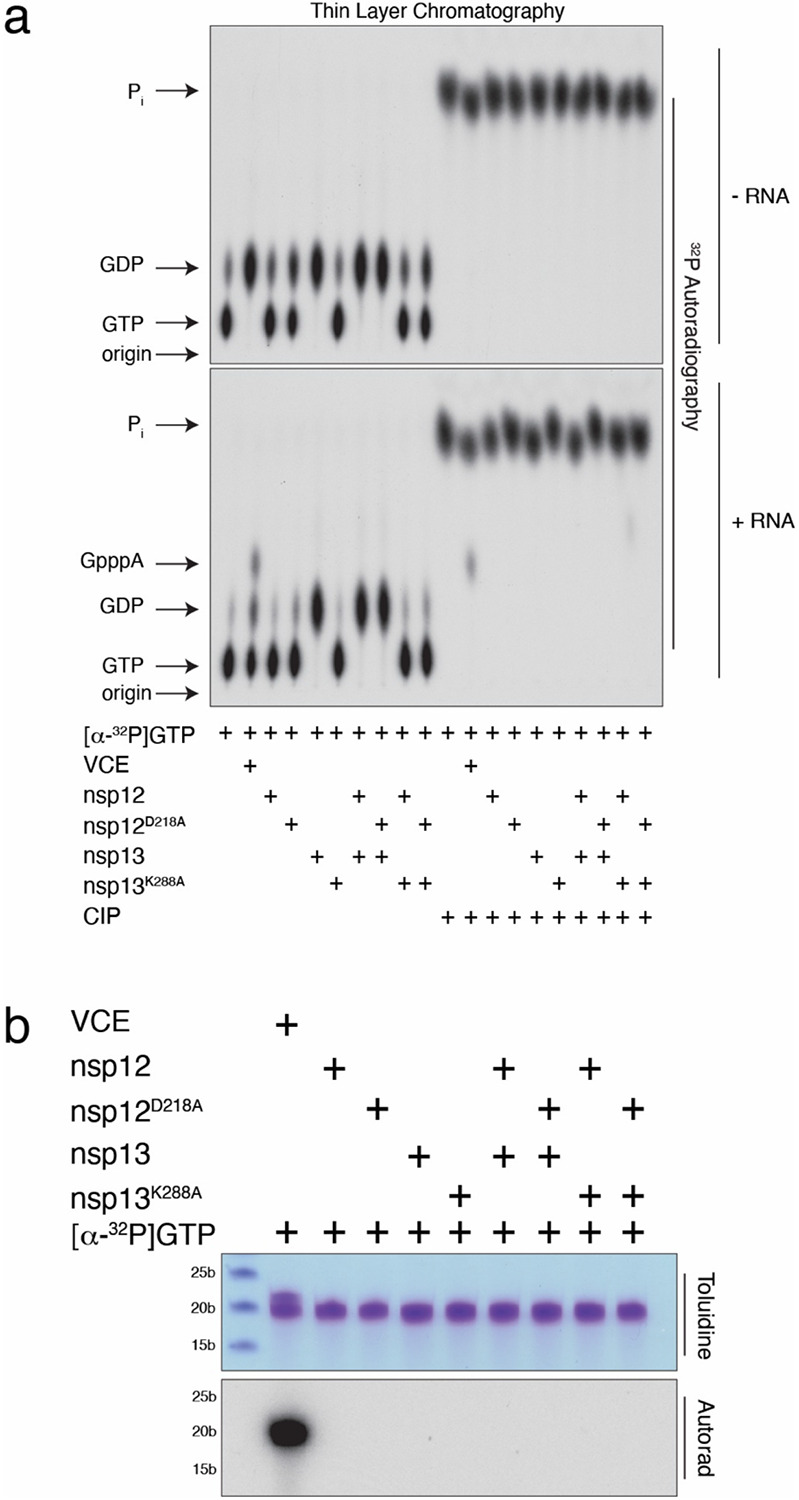
The NiRAN domain does not act as a GTase. **a.** Thin-layer chromatograms depicting the reaction products resulting from the incubation of [α-^32^P]GTP with nsp12 or the inactive NiRAN mutant (D218A). Reactions were performed as described in ^21^ with (*lower*) or without (*upper*) 5’-pppRNA^A19C^ and included nsp13 or the inactive mutant (K288A) as indicated. Vaccinia capping enzyme (VCE) was used as a positive control. Reaction products were digested with nuclease P1, then treated with or without calf intestinal alkaline phosphatase (CIP) and analysed by PEI-cellulose thin-layer chromatography (TLC) followed by autoradiography. The positions of the origin and standard marker compounds are indicated. **b.** RNA products from (**a)** were analysed by TBE Urea-PAGE and visualized by toluidine blue O staining (*upper*) and autoradiography (*lower*). Markers indicate RNA size by base length.

**Extended Data Table 1.** RNAs used in this study

RNA	Sequence
5′-pppRNA^A19C 21^	[ppp]ACCCCCCCCCCCCCCCCCCC
5′-pppRNA^LS10^	[ppp]AUUAAAGGUU
5′-pppRNA^LS2^	[ppp]AU
5′-pppRNA^LS3^	[ppp]AUU
5′-pppRNA^LS4^	[ppp]AUUA
5′-pppRNA^LS5^	[ppp]AUUAA
5′-pppRNA^LS6^	[ppp]AUUAAG
5′-pppRNA^LS20^	[ppp]AUUAAAGGUUUAUACCUUCC
5′-pppRNA^LS10_A1C^	[ppp]CUUAAAGGUU
5′-pppRNA^LS10_A1G^	[ppp]GUUAAAGGUU
5′-pppRNA^LS10_A1U^	[PPP]UUUAAAGGUU
5′-pppRNA^LS10_U2G^	[ppp]AGUAAAGGUU

**Extended Data Table 2.** Data collection and refinement statistics.

	
Magnification	81,000
Voltage (kV)	300
Electron exposure (e^−^Å^−2^)	54
Defocus range (μm)	−1.0 to −2.5
Pixel size (Å)	1.09
Symmetry imposed	C1
Initial particle images (no.)	4,196,806
Final particle images (no.)	39,985
Map resolution (Å)	3.2
FSC threshold	0.143
**Refinement**	
Initial model used (PDB code)	-
Model resolution (Å)	3.5
FSC threshold	0.5
Map sharpening *B* factor (Å^−2^)	−37
Nonhydrogen atoms	9,215
Protein residues	1150
Ligands	4
B factors (Å^−2^)	
Protein	67.4
Ligands	75.9
R.m.s. deviations	
Bond lengths (Å)	0.002
Bond angles (°)	0.453
MolProbity score	1.50
Clashscore	3.12
Poor rotamers (%)	0
Favored (%)	94.1
Allowed (%)	5.9
Disallowed (%)	0

**Extended Data Table 3.** Oligonucleotides used in the SARS-CoV-2 infection experiments

Oligo	Sequence	Use
PacI Forward	GGTTGAAGCAGTTAATTAAAGTTACACTTGTG	Fragment 1 and 3
1NtoA Reverse	GCAACAGGACTAAGCTCATTAGCCTGTAATTTGACAGC	Fragment 1
1NtoD Reverse	GCAACAGGACTAAGCTCATTGTCCTGTAATTTGACAGC	Fragment 1
2NtoA Reverse	GCAACAGGACTAAGCTCAGCATTCTGTAATTTGACAGC	Fragment 1
73KtoA Reverse	GAGAAAGTGTGTCTCGCAACTACAAAGTAAG	Fragment 1
MluI Reverse	CCTAAGTTGGCGTATACGCGTAATATATCTGGG	Fragment 2 and 3
1NtoA Forward	GCTGTCAAATTACAGGCTAATGAGCTTAGTCCTGTTGC	Fragment 2
1NtoD Forward	GCTGTCAAATTACAGGACAATGAGCTTAGTCCTGTTGC	Fragment 2
2NtoA Forward	GCTGTCAAATTACAGAATGCTGAGCTTAGTCCTGTTGC	Fragment 2
73KtoA Forward	CTTACTTTGTAGTTGCGAGACACACTTTCTC	Fragment 2
